# The BCL2 family: from apoptosis mechanisms to new advances in targeted therapy

**DOI:** 10.1038/s41392-025-02176-0

**Published:** 2025-03-21

**Authors:** Meike Vogler, Yannick Braun, Victoria M. Smith, Mike-Andrew Westhoff, Raquel S. Pereira, Nadja M. Pieper, Marius Anders, Manon Callens, Tim Vervliet, Maha Abbas, Salvador Macip, Ralf Schmid, Geert Bultynck, Martin JS Dyer

**Affiliations:** 1 Goethe University Frankfurt, Institute for Experimental Pediatric Hematology and Oncology, Frankfurt am Main, Germany; 2 German Cancer Consortium (DKTK) partner site Frankfurt/Mainz, a partnership between DKFZ and University Hospital Frankfurt, Frankfurt am Main, Germany; 3 University Cancer Center Frankfurt (UCT), University Hospital Frankfurt, Goethe University Frankfurt, Frankfurt am Main, Germany; 4 Department of Pediatric Surgery, University Hospital Frankfurt, Goethe University Frankfurt, Frankfurt am Main, Germany; 5 The Ernest and Helen Scott Haematological Research Institute, Leicester Cancer Research Centre, University of Leicester, Leicester, UK; 6 Department of Pediatrics and Adolescent Medicine, University Medical Center Ulm, Ulm, Germany; 7 KU Leuven, Lab. Molecular & Cellular Signaling, Dep. Cellular & Molecular Medicine, and Leuven Kankerinstituut (LKI), Leuven, Belgium; 8 Mechanisms of Cancer and Ageing Laboratory, Department of Molecular and Cell Biology, University of Leicester, Leicester, UK; 9 Josep Carreras Leukaemia Research Institute, Badalona, Spain; 10 FoodLab, Faculty of Health Sciences, Universitat Oberta de Catalunya, Barcelona, Spain; 11 Department of Molecular and Cell Biology, University of Leicester, Leicester, UK; 12 Institute for Structural and Chemical Biology, University of Leicester, Leicester, UK

**Keywords:** Senescence, Molecular medicine

## Abstract

The B cell lymphoma 2 (BCL2) protein family critically controls apoptosis by regulating the release of cytochrome c from mitochondria. In this cutting-edge review, we summarize the basic biology regulating the BCL2 family including canonical and non-canonical functions, and highlight milestones from basic research to clinical applications in cancer and other pathophysiological conditions. We review laboratory and clinical development of BH3-mimetics as well as more recent approaches including proteolysis targeting chimeras (PROTACs), antibody-drug conjugates (ADCs) and tools targeting the BH4 domain of BCL2. The first BCL2-selective BH3-mimetic, venetoclax, showed remarkable efficacy with manageable toxicities and has transformed the treatment of several hematologic malignancies. Following its success, several chemically similar BCL2 inhibitors such as sonrotoclax and lisaftoclax are currently under clinical evaluation, alone and in combination. Genetic analysis highlights the importance of BCL-X_L_ and MCL1 across different cancer types and the possible utility of BH3-mimetics targeting these proteins. However, the development of BH3-mimetics targeting BCL-X_L_ or MCL1 has been more challenging, with on-target toxicities including thrombocytopenia for BCL-X_L_ and cardiac toxicities for MCL1 inhibitors precluding clinical development. Tumor-specific BCL-X_L_ or MCL1 inhibition may be achieved by novel targeting approaches using PROTACs or selective drug delivery strategies and would be transformational in many subtypes of malignancy. Taken together, we envision that the targeting of BCL2 proteins, while already a success story of translational research, may in the foreseeable future have broader clinical applicability and improve the treatment of multiple diseases.

## Introduction

**Fig. 1:**
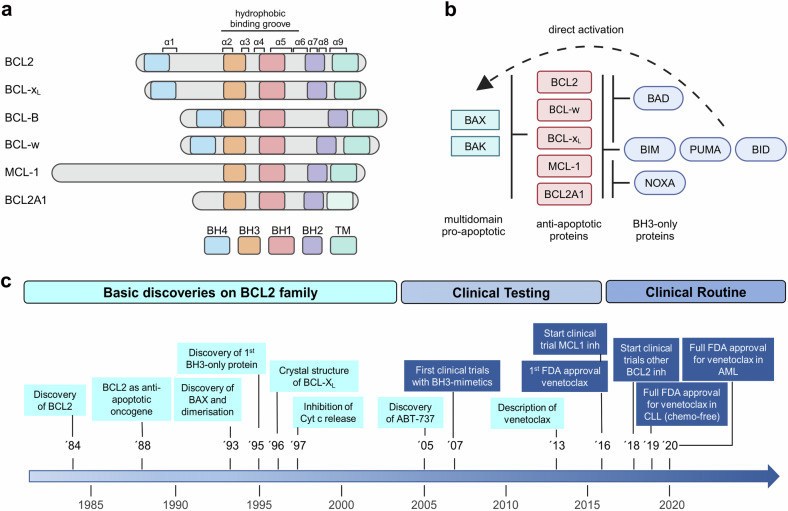
**Milestones in the development of BH3-mimetics**. **a** Overview of the anti-apoptotic BCL2 family proteins containing the BH domains and alpha helical structures α1-α9. **b** General interaction pattern within the BCL2 family, with the anti-apoptotic proteins inhibiting the pore-forming pro-apoptotic proteins BAX and BAK. The BH3-only proteins inhibit the anti-apoptotic BCL2 proteins and may also induce direct activation of BAX and BAK. **c** Timeline illustrating milestones and achievements in the discovery of BCL2 proteins and the development of BH3-mimetics. **a** and **b** Created in BioRender

The induction of cell death is one of the fundamental aspects regulating tissue homeostasis and counterbalancing proliferation. It is vital for developmental tissue sculpting such as digit formation, the elimination of auto-reactive immune cells and the removal of cells which are damaged beyond repair. Dysregulation of cell death can lead to pathological conditions associated with increased cell mass or tissue loss.^1^ The most prominent form of regulated cell death is apoptosis, which can be initiated via extracellular ligands (extrinsic apoptosis) or internal signals (intrinsic apoptosis). Several modes of cross-talk exist between the pathways and both require the activation of caspases to execute cell death. Intrinsic apoptosis is critically regulated by the BCL2 protein family.^2^ This protein family contains both pro- and anti-apoptotic family members that interact with each other to regulate the release of cytochrome c and other noxious proteins from the mitochondria into the cytosol. Once cytosolic, cytochrome c leads to formation of the apoptosome complex and activation of caspase-9 and the caspase cascade, ultimately leading to cell death.^3^ In humans, the BCL2 protein family contains about 20 proteins, that either act to facilitate or prevent apoptosis. Shared and defining characteristics of the BCL2 protein family are structural elements called BCL2 homology (BH) domains consisting of stretches of up to 15 amino acids.^4^ The BCL2 family proteins can be divided into three groups, based on function and number of domains: multi-domain anti-apoptotic proteins (BCL2, BCL-X_L_, BCL-w, MCL1, BCL2A1, BCLB), multi-domain pro-apoptotic proteins (BAK, BAX and BOK) and BH3-only pro-apoptotic proteins (BID, BIM, BAD, BIK, NOXA, PUMA, BMF and HRK) (Fig. 1a and b). Thus, this family can be regarded as a tripartite apoptotic switch. Cellular stress causes the upregulation or activation of pro-apoptotic BH3-only proteins which inhibit the anti-apoptotic proteins and activate the multi-domain pro-apoptotic proteins. The BH sequence motifs are evolutionary highly conserved and hence BCL2 proteins are not only found in mammals, but also in metazoans and several viruses.^5^ For the latter, expression of BCL2 genes may maintain survival of infected cells and counteract attack by the innate immune cells^6^ highlighting that a deregulated BCL2 family leads to disturbed apoptosis in the affected cells. In humans, dysregulation of the BCL2 protein family is associated with several diseases including cancer, neurodegenerative diseases and autoimmune diseases. In cancer, most malignancies retain dependence on one or more anti-apoptotic BCL2 proteins and these therefore are prime candidates for therapeutic targeting. Thus, the detailed mechanistic and structural understanding of the BCL2 protein family has paved the way for rationally designed targeting strategies, which are summarized and discussed in this review.

### Discovery of BCL2 proteins and their importance in apoptosis regulation

The founding and eponymous anti-apoptotic protein, BCL2, was discovered in 1984 as the gene involved in the t(14;18)(q32.3;q21.3) chromosomal translocation found in 85% of follicular lymphoma (FL).^7–9^ This translocation juxtaposes the *BCL2* gene on chromosome 18 with the immunoglobulin heavy chain (*IGH*) enhancer region on chromosome 14, which results in an overexpression of BCL2. Apart from FL, this chromosomal translocation has also been observed in diffuse large B cell lymphoma (DLBCL) and chronic lymphocytic leukemia (CLL).^10^

Following on its initial description, BCL2 was discovered to function as an inhibitor of apoptosis, representing the first example of an oncogene that contributes to cancer by blocking cell death rather than promoting proliferation^11^ (Fig. 1c). In 1993, BAX was discovered as BCL2 homolog, and based on the binding of BAX to BCL2, a first model of heterodimerization between anti- and pro-apoptotic BCL2 proteins was suggested.^12^ This was further supported by structural studies describing the hydrophobic groove as main protein-protein interaction site within the BCL2 family.^13,14^ The discovery of the first BH3-only proteins, BIK^15^ and BID,^16^ led to the identification of the BH3 domain as the main binding partner for the hydrophobic groove. More mechanistic insight into the regulation of apoptosis was gained in 1997 by the identification of cytochrome c release as the main apoptotic event blocked by BCL2.^17,18^ Over the last decades, the main principles of apoptosis regulation by the BCL2 protein family have been characterized, with several models attempting to explain the complex interactions within this protein family.^19–21^

### Milestones on the way to the first clinical compounds

The discovery of the hydrophobic groove on the surface of the anti-apoptotic BCL2 proteins^13^ and its importance for the binding to pro-apoptotic BCL2 family proteins^14^ has paved the way for the development of compounds that selectively bind into the hydrophobic groove and hence functionally neutralize the targeted anti-apoptotic BCL2 protein. While many early putative BH3-mimetics turned out to be unspecific and mainly induced apoptosis *via* endoplasmatic reticulum (ER) stress,^22,23^ the discovery of ABT-737 provided the first specific and potent tool compound for lab-based research.^24,25^ It was developed in 2005 using nuclear magnetic resonance (NMR)-based screening, parallel synthesis and structure-based design to inhibit BCL-X_L_. This technology is based on the linkage of proximally binding fragments to achieve specific and high-affinity binding.^26^ The discovery of ABT-737 represents one of the first successful attempts at targeting a protein-protein interface using a small molecule.

Modifications of ABT-737 led to the development of ABT-263 (navitoclax) with improved oral availability, which proceeded to clinical testing as described in detail below.^27,28^ Both ABT-737 and ABT-263 bind with nanomolar affinity to BCL2, BCL-X_L_ and BCL-w but not to MCL1 or BCL2A1, which display less homology in their hydrophobic groove.^29–31^ Similarity in the long hydrophobic groove of BCL2, BCL-X_L_ and BCL-w made the development of selective inhibitors more challenging, but in 2013 the first selective BCL2 inhibitor, ABT-199 (venetoclax) was generated.^32^ Rapid clinical development of venetoclax with highly promising results obtained in clinical studies^33–35^ led to a first FDA and EMA approval of venetoclax in 2016. The approval of venetoclax as a first-in-class BH3-mimetic and its clinical success in the treatment of leukemia showcase the impact that fundamental mechanistic research may have on patients’ lifes.

## The BCL2 protein network regulates apoptosis

### The importance of the anti-apoptotic BCL2 proteins in maintaining mitochondrial integrity

The release of cytochrome c from mitochondria into the cytosol is frequently recognized as a point of no return for cell death, and hence its regulation is essential for tissue homeostasis. In this context, the BCL2 proteins are key in regulating the mitochondrial outer membrane permeabilization (MOMP) leading to cytochrome c release. The BCL2 protein family comprises both pro- and anti-apoptotic proteins which may prevent or facilitate MOMP. Essential for the inhibition of MOMP are the six anti-apoptotic BCL2 proteins including BCL2 itself, BCL-X_L_ (*BCL2L1*),^36^ Bfl-1 (*BCL2A1*),^37^ BCL-w (*BCL2L2*),^38^ BCL-B (*BCL2L10*)^39^ and MCL1^40^ (Fig. 1a). These globular α-helical proteins share extensive sequence and structural similarity and contain four BH domains.^41,42^ Typically, there is an eight-helix bundle (encoded within the BH1, 2 and 3 domains) which forms a hydrophobic surface groove for the binding of BH3 domains of other BCL2 family members. Interactions are modulated by four hydrophobic pockets (P1-4) within the binding groove.^14^

**Fig. 2:**
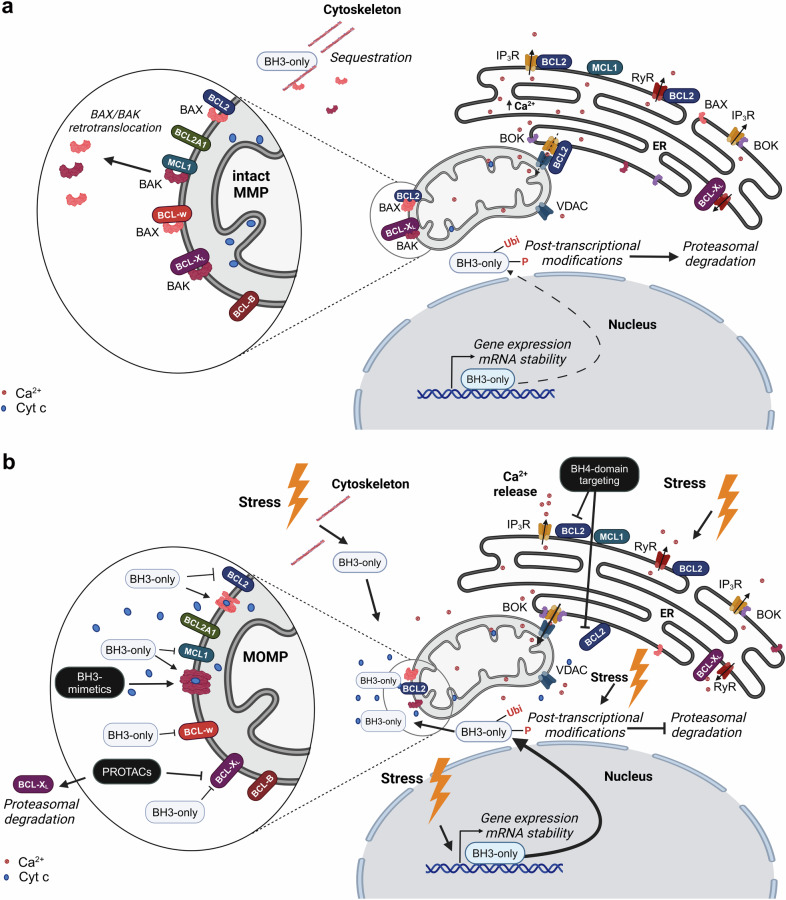
**Overview of the BCL2 family and the regulation of cytochrome c release**. **a** In unstressed healthy cells, several mechanisms, including canonical and non-canonical functions of BCL2 family members, prevent MOMP and release of cytochrome c from mitochondria, including the retrotranslocation of BAX/BAK and the limited availability of BH3-only proteins at mitochondrial membranes and preventing mitochondrial Ca^2+^ overload. **b** Upon cellular stress, BH3-only proteins become available at the mitochondria and facilitate BAX/BAK oligomerization, leading to MOMP. This is also facilitated by increased Ca^2+^ signaling including Ca^2+^ transfers from ER stores towards mitochondria. Created in BioRender

Another shared function of the anti-apoptotic BCL2 proteins is their ability to integrate into the outer mitochondrial membrane (OMM) *via* a C-terminal transmembrane (TM) domain and to interact with pro-apoptotic BCL2 family members residing both in the OMM and in the cytoplasm^43^ (Fig. 2). Localization at the OMM is key to the canonical function of anti-apoptotic BCL2 proteins, and even BCL2A1, which does not contain a well-defined transmembrane domain, is localized at the mitochondria of healthy cells.^44,45^

### Regulation of intracellular Ca^2+^ signaling at the ER

In addition to their mitochondrial localization, all anti-apoptotic BCL2 family members also reside at other compartments including the ER, the main Ca^2+^ storage organelle (reviewed in ref. ^46,47^). The steady-state ER Ca^2+^ levels as well as the ER Ca^2+^-release properties critically contribute to a broad variety of cell death processes, including apoptosis.^48^ Furthermore, the ER and mitochondria are closely connected to each other via ER-mitochondrial tethers, thereby enabling the efficient exchange of lipids, reactive oxygen species (ROS) and Ca^2+^ between ER and mitochondria.^49^ These ER-mitochondrial contact sites also harbor Ca^2+^-transport systems, including inositol 1,4,5-trisphosphate receptors (IP_3_R) at the ER, and voltage-dependent anion channels (VDACs) at the OMM.^50^ The inter-organellar Ca^2+^ exchanges between ER and mitochondria regulate cell death and survival by promoting cellular fitness and mitochondrial metabolism.^50–52^ Overload of mitochondrial Ca^2+^ triggers cell death by opening of the mitochondrial permeability transition pore.^48,53^ Several anti-apoptotic BCL2 family members protect against apoptosis by operating at the ER and influencing ER Ca^2+^ homeostasis and dynamics. Strong evidence for a key role for BCL2 at the ER came from BCL2-cytochrome B5 fusion strategies, which target BCL2 to the ER. The expression of BCL2-cytochrome B5 conferred apoptosis protection against a broad spectrum of cell death inducers (reviewed in ref. ^54^). Nowadays, it has become clear that anti-apoptotic BCL2 profoundly impacts ER Ca^2+^ handling in multiple ways. BCL2 can lower steady state ER Ca^2+^ levels, thereby indirectly limiting ER Ca^2+^ efflux and thus preventing mitochondrial Ca^2+^ overload.^55–57^ Via its N-terminal BH4 domain, BCL2 also directly suppresses the IP_3_R-mediated release of Ca^2+^ from the ER.^58,59^ Disrupting the IP_3_R/BCL2 complex using IP_3_R-derived, BH4 domain-targeting peptide-based decoys for BCL2 antagonizes BCL2’s protective effect against Ca^2+^-driven apoptosis.^60^ Several other anti-apoptotic BCL2 family members have also been reported to exert their anti-apoptotic function by intersecting with ER Ca^2+^ handling, controlling IP_3_Rs, and/or ER-mitochondrial Ca^2+^ exchanges,^61^ including BCL-X_L_,^62–65^ MCL1,^66^ BCL-B^67–69^ and BCL-w.^70,71^ In addition to IP_3_Rs, ryanodine receptors, another class of intracellular Ca^2+^-release channels mainly present at the ER in excitable cells, are also targeted by anti-apoptotic BCL2 family members.^72–74^ In particular, both BCL2 and BCL-X_L_ can bind to ryanodine receptors, thereby inhibiting their Ca^2+^-flux properties.

The importance of the individual anti-apoptotic BCL2 proteins is not only determined by their different molecular function, but also by their cell type- and tissue-specific expression. In this regard, some anti-apoptotic BCL2 proteins like BCL-w^75^ and BCL2A1^76^ are only expressed in certain tissues, while others like MCL1 appear to be more ubiquitously expressed. Of note, the expression patterns also display species differences, with e.g. BCL2A1 expression being restricted to the hematopoietic system in mice but more widespread expression in humans.^37,77^

### The BH3-only proteins as sentinels of cellular stress

The pro-apoptotic BCL2 family members comprise two main groups based on their structure and function (Fig. 1a, b): the multidomain pro-apoptotic BCL2 proteins BAX, BAK (*BAK1*) and BOK, and the BH3-only proteins, including BIM (*BCL2L11*), PUMA (*BBC3*), BID, NOXA (*PMAIP1*), BAD (*BCL2L8*), BMF, BIK, HRK (*DP5*) and some less well-established family members like proteins of the BNIP family.^78,79^ In contrast to the multidomain BCL2 proteins, the BH3-only proteins are intrinsically unstructured proteins.^80–83^ They only share the BH3 domain, which is unstructured in solution but folds into an alpha helix when interacting with a BCL2 binding partner.^29,80^ Structurally, BID differs from other BH3-only proteins since, similar to the multidomain BCL2 proteins, it contains a core structure and needs to be cleaved at an interhelical loop for exposure of the BH3 domain for interaction with its binding partners.^84–86^ Since cleavage of BID to tBID can be mediated by caspase-8 in the extrinsic apoptotic pathway, BID serves as an important crosstalk between extrinsic and intrinsic apoptotic pathways.^87^ In addition to their BH3 domain, some BH3-only proteins also contain a C-terminal anchor domain, allowing their insertion into membranes.^88,89^ Via their BH3 domain, BH3-only proteins may bind into the hydrophobic groove of the anti-apoptotic BCL2 proteins, thus forming heterodimers and preventing BAX and BAK sequestration.^90^ Based on the chemical nature of their BH3 domain, some BH3-only proteins like BIM, BID and PUMA can bind to all anti-apoptotic BCL2 proteins, while others interact only with selected binding partners offering high affinity binding to the hydrophobic groove.^91,92^ BH3-only proteins can also be functionally divided into direct activators or sensitizers. Thereby, the direct activator BH3-only proteins BIM, tBID and PUMA may directly bind to the multidomain pro-apoptotic proteins BAX and BAK to facilitate their pore-forming ability.^93,94^ In contrast, the sensitizer BH3-only proteins only interact with anti-apoptotic BCL2 proteins and counteract their sequestration of BAX and BAK^95^ (Fig. 1b).

Given their role as stress sensors in a cell, the activity of BH3-only proteins is tightly regulated by multiple different mechanisms, including transcriptional regulation, protein stability, phosphorylation and accessibility at the mitochondria. Some BH3-only proteins are more constitutively expressed and kept in check by sequestration at cytoskeletal proteins, like dynein and myosin complexes, as shown for BIM and BMF.^96,97^

### Mitochondrial perturbations mediated by BAX and BAK

The multidomain pro-apoptotic proteins BAX and BAK share an overall α-helical structure with the anti-apoptotic BCL2 proteins containing a C-terminal TM domain and all four BH domains which comprise a hydrophobic pocket.^98^ In addition to their ability to integrate into the OMM, they can oligomerize within the OMM and form macromolecular pores.^12,99^ Thus, BAX, BAK and to a less well-characterized extent also BOK are responsible for MOMP and the release of cytochrome c into cytosol. Their oligomerization within the OMM may be inhibited by sequestration by the anti-apoptotic BCL2 proteins *via* their hydrophobic groove.^31^ Thus, by binding to BAX and BAK, the anti-apoptotic BCL2 proteins are essential in preventing MOMP. The function of BOK in apoptosis regulation is less clear, with some studies suggesting a more prominent role of BOK at the Golgi and ER membranes.^100–102^

The events leading to the activation and oligomerization of BAX and BAK are in parts shared between both proteins. Both BAX and BAK can shuttle between a cytosolic location and an association with the OMM. BAX is mainly cytosolic in unstressed cells and undergoes constant retrotranslocation to the cytosol, thus preventing toxic accumulation of BAX at the OMM.^103^ In contrast, BAK is mainly mitochondrial even in healthy cells and undergoes cytosolic retrotranslocation at much slower rates than BAX.^104^ For apoptosis initiation, both BAX and BAK must undergo a series of conformational changes, which are initiated by interaction with BH3-only proteins upon cellular stress and lead to the accumulation of BAX/BAK at the mitochondria.^105,106^ BH3-only proteins can either interact with BAX and BAK at the hydrophobic groove, or alternatively *via* a rear activation site presented by α1-α6 helices.^93,107^ Ultimately, the conformational changes of BAX and BAK result into the exposure of their BH3 domain which enables dimerization via a BH3-in-groove interaction.^108,109^ Of note, while heterodimers of BAX and BAK have been observed, their occurrence is less prevalent than homodimers of either BAX or BAK, which may be explained by lower binding affinity and less compatibility for the opposite BH3 domains.^110,111^ At the lipid interface presented by the OMM, BAX or BAK dimers can assemble into higher-order complexes.^112^

The precise nature of the pore formed by BAX and BAK in the OMM has been a key question for the last decades, and advances in biophysics and superresolution microscopy have shed some light on the pore structures.^113,114^ Recent evidence supports a toroidal pore model characterized by the fusion of the outer and inner leaflets of the OMM.^115^ In the toroidal pore, a continuous surface is formed that consists of lipids and proteins and allows membrane permeabilization to facilitate the release of intermembrane space proteins. To this end, BAX oligomers have been shown to assemble into large ring-like structures, as well as arcs and lines.^116–118^ Pore formation in the OMM may be triggered by shallow insertion of BAX or BAK into the outer leaflet and focal assembly of oligomers, resulting in increased membrane tension and curvature. This model also implies a potential function of BAX and BAK in mitochondrial membrane remodeling, as observed previously.^119–121^ In line with this, next to their role in mediating MOMP, active BAX, and BAK also play a key role in shaping the mitochondria and influencing the rate of fission and fusion.^120,122^ Therefore, BAX and BAK may be regarded as key regulators of mitochondrial dynamics.

In addition to their mitochondrial function, BAX and BAK also operate in the ER. Cells deficient in BAX/BAK display a decreased ER Ca^2+^-store content,^123^ which contributes to apoptosis resistance.^57^ Restoring ER Ca^2+^ levels by overexpressing the ER Ca^2+^-uptake pump SERCA increased cell death in BAX/BAK-deficient cells.^123^ How BAX and BAK impact ER Ca^2+^ stores remains not fully understood. One contributing mechanism is an increased ER Ca^2+^ leak *via* Protein Kinase A-mediated phosphorylation of IP_3_Rs, which renders the channels hypersensitive to their ligand IP_3_.^124^ In addition, in the absence of BAX/BAK, anti-apoptotic BCL2 proteins may become available for other targets such as the protein BAX Inhibitor-1/TMBIM6,^125,126^ which has been proposed as an ER Ca^2+^ leak channel that operates downstream of BCL2.^127^ BAX/BAK also function at ER-mitochondrial contact sites. During apoptosis, BAX appears to be recruited to these organellar contact sites, which become stabilized by sumoylation of DRP1.^128^ These ER-mitochondrial contact sites serve as platforms to recruit the cell death machinery and inter-organellar Ca^2+^ fluxes, which drive cytochrome c release.

Besides BAX and BAK, BOK also operates at the ER. In contrast to BAX/BAK, BOK, which appears to be constitutively active, has been proposed to be mainly controlled through protein turnover. BOK is continuously ubiquitylated with subsequent proteasomal degradation via ER-associated degradation.^102^ Furthermore, BOK is strongly associated to IP_3_R channels.^129^ BOK scaffolded to these channels is stabilized and protected from proteolytic degradation. The recruitment of BOK to IP_3_Rs also counteracts BOK’s pro-apoptotic activity. In addition to this, BOK stabilizes ER-mitochondrial contact sites and promotes ER-mitochondrial Ca^2+^ exchanges, thereby contributing to its pro-apoptotic effect.^130^ In BOK-deficient cells, expression of BOK mutants that fail to bind to IP_3_Rs support ER-mitochondrial contact site formation but fail to restore inter-organellar Ca^2+^ fluxes and apoptosis. Other reports however indicated that BOK mainly affects mitochondrial fusion rates, enhancing mitochondrial fragmentation, though without impacting IP_3_R-mediated Ca^2+^ release or mitochondrial Ca^2+^ transfers.^131^

## Regulation of BCL2 proteins in physiological and pathological conditions

### Control of embryonal development and organogenesis by BCL2 proteins

Due to their central function in apoptosis regulation, BCL2 proteins are key for balanced tissue generation during embryonal development. Early studies performed in knockout mice indicated that the deletion of *BCL2* family genes affects multiple organs and tissues. *BCL2* knockout animals displayed growth retardation and early postnatal mortality^132^ and *BCL2L1* or *MCL1* knockout mice were embryonically lethal.^133,134^ Even mice with combined deletion of single alleles of *MCL1* and *BCL2L1* were unable to survive and died at birth with multiple severe defects, suggesting overlapping functions of MCL1 and BCL-X_L_ during embryonic development.^135^ Taken together, these data confirm that these anti-apoptotic genes are essential in embryonic development. Furthermore, lineage-selective deletion of different anti-apoptotic BCL2 proteins demonstrated that different types of non-transformed, differentiated cells rely on anti-apoptotic BCL2 proteins.^136–141^ For example, the BCL2 proteins play a crucial role in neuronal survival and apoptosis regulation within the central nervous system (CNS). The expression of BCL2 proteins varies between developing and mature neurons and between different brain regions, highlighting an important function of the BCL2 proteins in brain development.^142^ This is further supported by a study showing that high expression of BAX in neurons regulates microglia maturation and shapes the brain milieu during embryogenesis.^143^

Mutation of BCL-w/BCL2L2 in a mouse strain resulted in male sterility and testicular degeneration associated with loss of Sertoli cells, highlighting an important role of BCL-w in testicular development and spermatogenesis.^144,145^ In addition, a role for BCL-w in B cell survival was reported, with loss of BCL-w leading to increased apoptosis upon growth factor withdrawal in B cells.^146^ Knockout of *BCL2A1* has been technically challenging due to quadruplication of the gene locus in mice, which complicated conventional knockout studies. While a constitutive knockdown of *BCL2A1* by RNAi indicated an essential function in multiple hematopoietic cell types,^147,148^ the development of a mouse strain devoid of all *BCL2A1* variants showed only a minor impact of BCL2A1 on cellular survival selectively in conventional dendritic cells.^149,150^ To the best of our knowledge, a knockout model for the remaining anti-apoptotic family member BCL-B has not yet been described, and its role in embryonal development is unclear.

The genetic deletion of the main pore-forming proteins *BAX* or *BAK* resulted in surprisingly minor phenotypes. Despite some morphological abnormalities, the mice were found to be developmentally normal and viable.^151^ However, combined deletion of *BAX* and *BAK* resulted in the perinatal death of most mice. This highlights the overlapping function of BAX and BAK and their ability to functionally compensate loss of only one pore-forming protein.^152^ Surviving double knockout mice showed multiple developmental defects, including the persistence of interdigital webs and accumulation of excess hematopoietic cells leading to autoimmune phenotypes. Additional deletion of BOK in a hematopoietic reconstitution model showed only a mild increase in lymphocyte counts, suggesting some functional redundancies also between BAX/BAK and BOK.^153^ Interestingly, in many tissues of surviving BAX/BAK double knockout mice, cell death still occurred at a normal rate. Although cells deficient of BAX and BAK are resistant to apoptosis, induction of autophagy may represent an alternative way to die for cells deficient in BAX and BAK, highlighting the interconnection of the core apoptotic machinery with other non-apoptotic cell death pathways.^154,155^

Given their partially overlapping functions, the genetic deletion of individual BH3-only proteins is generally well tolerated in mice and the observed phenotypes are relatively mild. An exception was observed upon homozygous deletion of *BIM*, which resulted in embryonic lethality in half of the animals and indicated a non-redundant function of BIM during embryogenesis.^156^ The function of most BH3-only proteins only became apparent upon induction of cell death, e.g. by withdrawal of cytokines or upon irradiation. Loss of *BBC3*/PUMA did not affect the survival of mice but thymocytes isolated from knockout mice underwent less apoptosis upon DNA damage.^157^ Similarly, *PMAIP1/NOXA* knockout mice showed no overt phenotype, but the response to stress was affected in a cell type- and stimuli-dependent manner.^158,159^ Of note, combined knockout of multiple BH3-only proteins had a much more severe phenotype, with loss of BIM and PUMA or BMF leading to autoimmunity in several organs.^160–162^ The combined deletion of BID, BIM and PUMA resulted in a phenotype similar to BAX/BAK double knockout mice.^163^ Taken together, these studies highlight the importance of BH3-only proteins in regulating BAX/BAK activation and subsequent apoptosis induction, particularly in immune cell homeostasis.

### Epigenetic and transcriptional regulation of BCL2 proteins

The expression of the BCL2 proteins is also regulated transcriptionally and post-transcriptionally.^164–166^ In contrast to mutations, epigenetic modifications are reversible and allow for quick changes of the expression of whole sets of genes. The epigenetic landscape is influenced by several epigenetic enzymes, such as histone acetyl-transferases and their counterpart histone deacetylases (HDAC) or DNA methyltransferases (DNMT). Hypermethylation of tumor suppressor genes or hypomethylation of oncogenic loci represent attractive targets for therapeutic approaches *via* targeting DNMTs or histone-modifying enzymes.^167,168^ Azacitidine and 5-aza-2’-deoxycytidine (decitabine) are clinically approved DNMT inhibitors that improved the treatment of myelodysplastic syndrome (MDS), chronic myelomonocytic leukemia (CMML) and AML.^169,170^ Several compounds have been applied in clinical studies as single agents, but also in combinations with small molecule inhibitors or chemotherapy. The epigenetic changes often prime the cells to be more susceptible for additional cytotoxic treatments and may reverse acquired resistance.^166,171–173^ Most DNMTs impact the BCL2 proteins by means of upregulation of the BH3-only protein NOXA, as shown for several cancer types.^174–176^ Pro-apoptotic BMF and anti-apoptotic BCL2A1 are also regulated through HDAC inhibitors.^177^ The expression of MCL1 is additionally regulated by methylation status, which correlates with cisplatin sensitivity in osteosarcoma.^178^

Readers of acetylation, such as BET proteins, influence the transcription of genes by enabling transcription elongation at promoters or enhancer regions, especially at super-enhancers. These often regulate important cellular processes, like proliferation, metabolic or cell death regulation^179^, and the BCL2 protein family is often differentially regulated in response to BET inhibitor treatment in several cancer types.^180–182^ As with “methylation-related” enzyme inhibitors, BETi are in ongoing clinical trials as single agents, but more often as combination therapy. To date, it is not been fully investigated which of the “epi-drugs” directly influence the expression of BCL2 family members or whether the impact on prominent regulatory pathways, such as NFκB, p53, or STAT, to name some are the main cause for changes in the expression of the BCL2 proteins.

#### Gene expression regulation of the anti-apoptotic BCL2 proteins

The chromatin status directly influences the transcription of BCL2 family genes through recruitment of transcription factors. Expression of BCL-X_L_ was increased by histone 3 Lys27 acetylation at the *BCL2L1* promoter which determined the occupancy by p300 and the transcription factor Ets-1 to induce transcription.^183^ Also, upstream survival signaling strongly influences the expression of BCL2 proteins. Several anti-apoptotic BCL2 proteins are well-described targets of STAT and Rel/NF-κB transcription factors.^184–188^ In addition, the Forkhead box O (FOXO) family of transcription factors induced by PI3K-AKT signaling influenced BCL-X_L_ and BCL2 expression.^189,190^ As a short-lived protein, MCL1 is strongly influenced by transcriptional regulation and induced by several transcription factors, such as HIF-1, Elk4, ATF4 or c-Myc, among others.^191–193^ E2F1 on the other hand led to transcriptional repression of MCL1.^194^

In addition to the chromatin status and upstream survival signaling, gene expression can be influenced by micro RNAs (miRNAs) or long non-coding RNAs (lncRNAs). The role of miRNAs can be oncogenic as well as tumor-suppressive, and many miRNAs are differentially expressed between healthy controls and cancerous tissues (reviewed in ref. ^195^). Several miRNAs targeting the anti-apoptotic BCL2 proteins have been identified in different diseases.^165^ BCL2 itself can be dysregulated *via* the downregulation or deletion of miR-15/16, microRNAs that suppress translation of multiple proteins over and above BCL2. Downregulation of miR-15/16 has been observed in CLL, prostate cancer, pituitary adenomas, and mesothelioma, thus contributing to high BCL2 expression in these malignancies.^196–200^ In leukemic cells, miR-145 downregulates BCL2 and simultaneously induces BAX expression.^201^ Additionally, the miRNAs themselves are influenced by lncRNAs that act as molecular sponges and thereby hinder the repressing effect of miRNAs.^202^

Furthermore, protein levels are influenced by mRNA stability. The stability of the *BCL2* mRNA was increased by RNA binding protein nucleolin.^203^ The mRNA binding protein HuR promoted protein stability of BCL2, MCL1, and BCL-X_L_.^204^ Another RNA binding protein and ribonuclease, Regnase-1 (also known as *MCPIP-1*), on the other hand, inhibited the expression of anti-apoptotic genes such as *BCL2L1*, *MCL1* or *BCL2A1* by cleavage of the corresponding mRNA.^205,206^

#### Epigenetic regulation of pro-apoptotic BCL2 proteins

The activity of the BH3-only proteins is tightly regulated and induced by cellular stress.^207^ Modes of regulation include transcriptional induction, post-translational modifications like ubiquitination and phosphorylation, or subcellular compartmentalization. PUMA and NOXA are transcriptionally induced by p53, and hence they can exert their pro-apoptotic functions in multiple stress situations that lead to the activation of p53.^159,208^ In a similar manner, several other transcription factors, including E2F-1,^209,210^ HIF-1a^211^ and FOXO1/3,^212–216^ induced the expression of multiple BH3-only proteins. Additionally, BIM was described to be regulated through the transcription factors IRF4,^217^ the non-classical AP1 family member BATF^218^ or the EMT-inducing factor ZEB1.^219^ On the other hand, several regulators associated with pro-survival signaling have been described to silence and inactivate the expression of BH3-only proteins, thus ensuring cellular survival.^220,221^

Besides the induction of transcription, the mRNA stability of BH3-only proteins is highly regulated and may be affected by miRNAs or molecular chaperones.^222–224^ Several miRNAs have been described to be involved in the regulation of BIM in various diseases, e.g. the miR-17-92 cluster that is overexpressed in aggressive hematological malignancies.^225^ MiR-130a also suppresses BIM, however, in osteoarthritis the lncRNA CIR is upregulated, leading to inhibition of miR-130a and increased BIM expression.^226^ In AML the lncRNA MORRBID is overexpressed and related to poor overall survival, as it directly controls transcription at the BIM promoter and keeps the gene poised.^227,228^ In the case of PUMA, the lncRNA NEAT1 induced EZH2-mediated histone methylation on a promoter region of miR-139. In turn, the miRNA was suppressed, resulting in increased PUMA levels.^229^ In colorectal cancer cells, hypermethylation prevented the transcription of the lncRNA TSLC8 through FOXO1, which prevented TSCL8 to act as a tumor suppressor by stabilizing pro-apoptotic PUMA.^230^ In contrast, miR-221 is known to reduce PUMA mRNA and protein expression.^231^ Results in hypoxia cell models indicate that the lncRNA GAS5 can sponge miR-221 and thereby influence PUMA expression.^232^ NOXA expression is also influenced by several miRNAs that can either act inhibitory such as miR-21,^233^ miR-200b^234^ or miR-197,^235^ or induce NOXA expression like miR-23a.^236^ Furthermore, NOXA is impacted through the regulation by lncRNAs. These studies highlight the interconnectivity between chromatin state, lncRNAs, and miRNAs in the regulation of BCL2 protein expression. In colorectal cancer the c-Myc/miR-1271-5/NOXA/MCL1 axis can be targeted through BET inhibitor treatment in combination with BH3-mimetics to induce apoptosis, highlighting the interplay of many epigenetic mechanisms in one treatment.^237^ Often, miRNAs also target transcription factors or proteins of signaling pathways, whose dysregulation in turn affects the expression of BCL2 family genes. The proto-oncogene Bmi1 for example is upregulated in mantle cell lymphoma and a target of miR-16-1. Bmi1 furthermore directly inhibits NOXA and BIM expression.^238^

### Phosphorylation and control by kinase signaling pathway

There are four major mechanisms by which signal transduction is regulated on the protein level: expression, localization, phosphorylation, and degradation. The phosphorylation of BCL2 family proteins is an underexplored topic, with a decreasing number of articles published on it in recent years. These often focus on the effect of a direct phosphorylation by the survival cascade PI3K/AKT on BCL2-mediated apoptosis,^239^ but even here the matter is not straightforward. BAD^240,241^ and BAX^242,243^ are direct targets of AKT, which thereby inhibits their pro-apoptotic function at the mitochondria. However, this signaling cascade also affects the BCL2 family via protein transcription.^244,245^

The other pathway that plays a central role in BCL2 family phosphorylation is the mitogen-activated protein (MAP) kinase signaling cascade. Here, different members of the signaling cascade, such as MAPK and JNK, can have opposite effects on the BCL2 family.^246^ JNK phosphorylates BIM and BMF at their conserved Thr112 sites, as well as Ser65 of BIM.^246^ This induces their translocation from the dynein and myosin V motor complexes to the mitochondria, where they contribute to apoptosis induction.^247^ Of note, also here the kinase might affect BIM protein expression. ERK1/2 also mediates the phosphorylation of BIM_EL_ at Ser65, as well as Ser55 and Ser73, leading to its disassociation from MCL1 and BCL-X_L_ and degradation, allowing MCL1 and BCL-X_L_ to exert their anti-apoptotic function, for example after serum withdrawal.^248^ Indeed, phosphorylation of the BCL2 family proteins often mediates protein disassociation, as it has also been described for BAD/BCL-X_L_, where MAP2K-mediated phosphorylation of BAD disrupts BAD/BCL-X_L_ interactions.^249^ Interestingly, phosphorylation of the BCL2 family often occurs in unstructured regions and might not be sufficient to prevent binding of pro-apoptotic factors, but instead creates binding sites for other proteins.^248^ This has been demonstrated for BAD/BCL-X_L_ complexes, where BAD can be phosphorylated at different sites by AKT, MAPK-activated kinase RSK, and cAMP-dependent protein kinase.^240,250,251^ Phosphorylation by the latter at Ser155 has been clearly demonstrated to lead to disassociation from BCL-X_L_ and promote its interaction with 14-3-3 proteins, which sequester BAD away from BCL-X_L_.^251^ In contrast, phosphorylation can also lead directly to protein degradation, as shown for MCL1. When mitotic cell death is induced, cyclin-dependent kinase 1 and several other kinases can phosphorylate MCL1 leading to its degradation. Interestingly, the same events also lead to BCL2 and BCL-X_L_ phosphorylation, which does not affect their protein levels but weakens the interaction with BAX and other pro-apoptotic proteins.^252^

Not surprisingly, the best-studied member of this family in terms of regulation by phosphorylation is the BCL2 protein itself. The first report showing that phosphorylation of BCL2 correlates with survival was published in 1994.^253^ Through a series of elegant experiments, it was determined that Ser70 in the loop region of the protein was the main target of growth factor agonist-initiated phosphorylation mediated by protein kinase C.^254^ Later, additional kinases that phosphorylate BCL2 at the mitochondria and facilitate survival were identified, such as the stress kinase SAPK, MAPKs and ERK1/2. Taken together, the phosphorylation of Ser70 is a highly dynamic process that can be mediated *via* several signaling cascades and is antagonized by a PP2A phosphatase.^254^ Phosphorylated BCL2 is not a good target for caspase-mediated cleavage and does not interact well with the pro-apoptotic family members, while BCL2 de-phosphorylated by PP2A is degraded by the proteasome, possibly at the mitochondria and ER.^255,256^ Additionally, a second phosphorylation pattern was identified, induced by prolonged exposure to antimitotic agents and possibly associated with increased susceptibility to cell death, indicating an opposite function as Ser70 phosphorylation.^254^ Here, additional residues found in the loop region are phosphorylated on top of Ser70, among them Thr69 and Ser87. This hyperphosphorylation also seems to occur at the ER, where it affects the Ca^2+^ dynamics.^257^

In summary, the regulation of BCL2 family proteins via phosphorylation seems to control their signaling via three independent routes: 1. Indirectly, *via* (in)activation of gene expression, thus affecting protein expression; 2. directly, *via* affecting the ability to dimerize with other family members and sequestration partners, and 3. directly, by facilitating proteasomal degradation of BCL2 family proteins and, thus again, affecting their protein expression.

### Proteasomal regulation of BCL2 proteins

Continuing from the previous argument, BCL2 localized at the ER membrane interacts rather stably with PP2A and blockage of this phosphatase led to proteasome-mediated degradation of BCL2.^256^ Interestingly, only BCL2 phosphorylated at Ser87 by MAPK and not phosphorylated by AKT, protein kinase C or cyclic AMP-dependent protein kinase, affected its proteasomal degradation.^258^ In addition to phosphorylation after specific stimuli, there is thus another level of regulation by the proteasome. Some BH3-only proteins like NOXA display a very short half-life of less than 2 hours and are rapidly degraded by the proteasome. Therefore, proteasome inhibitors were found to induce NOXA protein expression independently of p53 activity.^259–261^ In addition, BIM, BIK, and BAX were also found to be regulated by proteasomal degradation.^262–264^ This implies that the stability of these BH3-only proteins is regulated by post-translational modifications like ubiquitination or NEDDylation. In line with this, the E3 ubiquitin ligase CHIP has been reported to control NOXA stability.^265^ Another interesting target of proteasomal degradation is MCL1, which is often found upregulated in a compensatory fashion when BCL2 is therapeutically inhibited.^266,267^ Upon cellular stress MCL1, in a stable complex with NOXA at the mitochondria, is phosphorylated by CDK2 at Ser64 and Thr70 and thus primed for degradation.^268^ At least four ubiquitinases and two deubiquitinases so far have been identified as regulating MCL1 degradation, suggesting that this is a tightly regulated process.^269,270^ This finding was initially thought to open up new potential targeting strategies in which MCL1 may be indirectly targeted by CDK inhibitors, like alvocidib and dinaciclib. However, initial clinical trials produced rather discouraging results.^186^ Taken together, it is becoming clear that phosphorylation of BCL2 proteins upon specific stimuli can lead to their proteasomal degradation and shift the balance between pro- and anti-apoptotic members of the family. However, additional mechanisms regulate the half-life of short-lived family members, leading to a complex regulation of BCL2 proteins in a cell type- and situation-dependent manner.

## BCL2 proteins as drug targets in cancer

### BCL2 family dependencies in cancer

The mechanistic understanding of the protein interactions within the BCL2 family and their functional consequences on the induction of apoptosis highlights the importance of the anti-apoptotic BCL2 proteins for cancer development. For cancer cells to survive their hostile environment, the anti-apoptotic BCL2 proteins must manage to keep the BH3-only proteins in check to prevent BAX/BAK pore formation and MOMP. Therefore, it is not surprising that many cancer cells display genetic alterations leading to increased expression of one or multiple anti-apoptotic BCL2 proteins.

**Fig. 3:**
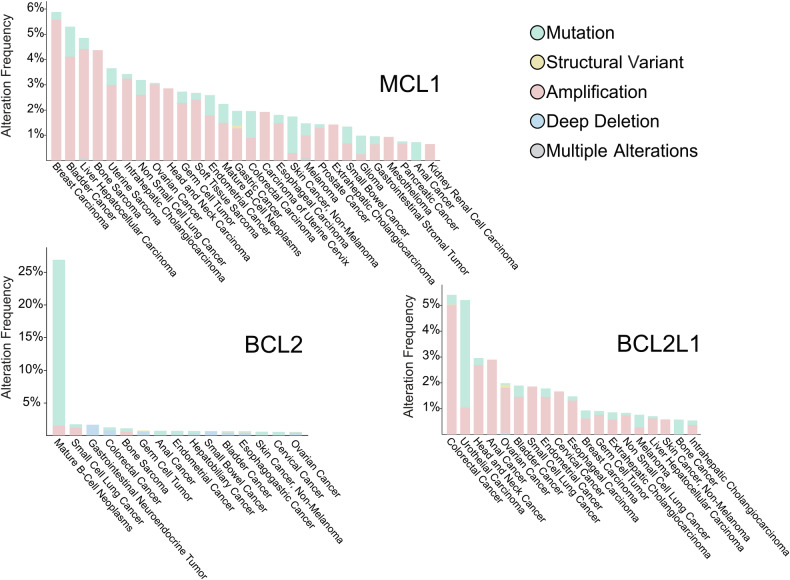
**Genetic alterations in cancer**. Analysis of genetic modifications involving *BCL2*, *BCL2L1* and *MCL1* using cBioportal^541–543^ was performed. Four main pan-cancer studies using targeted deep sequencing and encompassing 71,060 samples were selected (MSK-IMPACT,^544^ Cancer Therapy and Clonal Hematopoesis,^545^ China Pan-Cancer^546^ and MSK MetTropism^547^) and analyzed for genetic alterations involving *BCL2*, *BCL2L1* or *MCL1* in different cancer types. Thresholds were set for 100 samples / cancer type and a minimal frequency of 0.5%

Some striking differences are observed in the frequency and nature of genetic events involving the anti-apoptotic *BCL2* genes (Fig. 3). In particular BCL2 is affected differently in comparison with BCL-X_L_ (encoded by *BCL2L1*) or MCL1. Apart from its involvement in chromosomal translocations,^7,9^ *BCL2* can either be mutated or amplified in B cell malignancies, pointing to an important contribution of BCL2 in B cells. In both FL and DLBCL, the t(14;18)(q32.3;q21.3) translocation is an initiating event in disease pathogenesis, allowing survival of cells otherwise destined to die in the germinal center. In other malignancies however, *BCL2* is rarely amplified and in complete contrast, *BCL2* is homozygous deleted in about 5% of colorectal cancer cases. This may be explained by its genetic proximity to the tumor suppressor gene Deleted in Colon Cancer (*DCC*) which lies 11 Mb centromeric of *BCL2* on the long arm of chromosome 18.^271,272^ *BCL2L1* and *MCL1* on the other hand are commonly (about 5% of cases) amplified in solid tumors; *BCL2L1* in colorectal cancer, while *MCL1* is most commonly amplified in breast cancer. Overall, *MCL1* is amplified at higher frequencies than *BCL2L1*, in line with a previous landmark paper describing copy number alterations of *MCL1* in up to 10% of patients for some cancer types.^273^

**Fig. 4:**
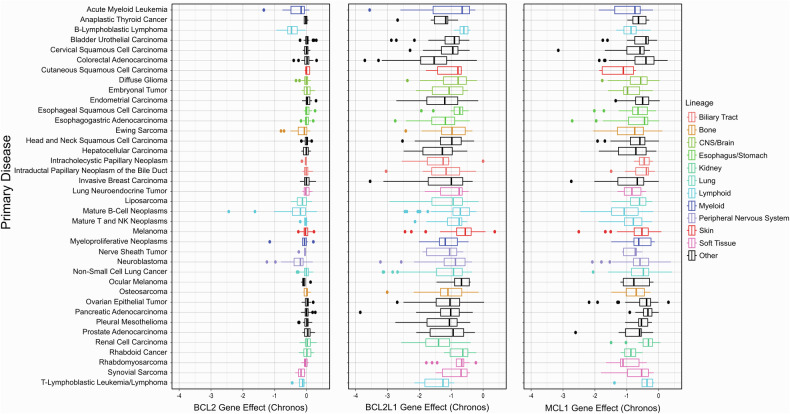
**BCL2 family dependencies in cancer**. Boxplots depicting the Chronos dependency scores of *BCL2*, *BCL2L1* and *MCL1* of cancer cell lines according to the DepMap data. Cancer subtypes (primary disease) with data available for *n* ≥ 5 cell lines were included. If multiple subtypes belonged to one lineage, the lineage was color-coded, the rest are shown in black

Analysis of cellular dependencies using large-scale CRISPR screens provided in Depmap (https://depmap.org/portal/)^274^ reveals some striking observations (Fig. 4). Only a few malignancies depend on BCL2 for survival (mean Chronos Gene Dependency Score −0.0462), most notably B cell malignancies, AML, and neuroblastoma. *BCL2L1* and to a lesser extent also *MCL1* are classified as common essential genes (mean Chronos Gene Dependency Scores −1.11 and −0.673, respectively), with only some cancer types being unaffected by deletion of either *BCL2L1* or *MCL1*. In line with its frequent amplification in colorectal malignancies, deletion of *BCL2L1* is highly lethal in colorectal cancer. Of note, many colorectal cell lines displaying *BCL2L1* dependency derive from rectal tumors, e.g. SW1463, SNU254, and C99. The most pronounced effect of *MCL1* deletion was observed in cutaneous squamous cell carcinoma, rhabdomyosarcoma, and mature B cell neoplasms. Taken together, these data highlight that the anti-apoptotic BCL2 proteins BCL2, BCL-X_L_, and MCL1 are all highly promising therapeutic targets in multiple cancer types. In contrast to these three main anti-apoptotic BCL2 proteins, the genes for the related anti-apoptotic BCL2 proteins Bfl1 (*BCL2A1*), BCL-w (*BCL2L2*) and BCL-B (*BCL2L10*) display little perturbation effects in cancer (mean Chronos Gene Dependency Scores −0.0454, −0.103 and 0.006, respectively).

### Targeting of BCL2 proteins with BH3-mimetics

**Fig. 5:**
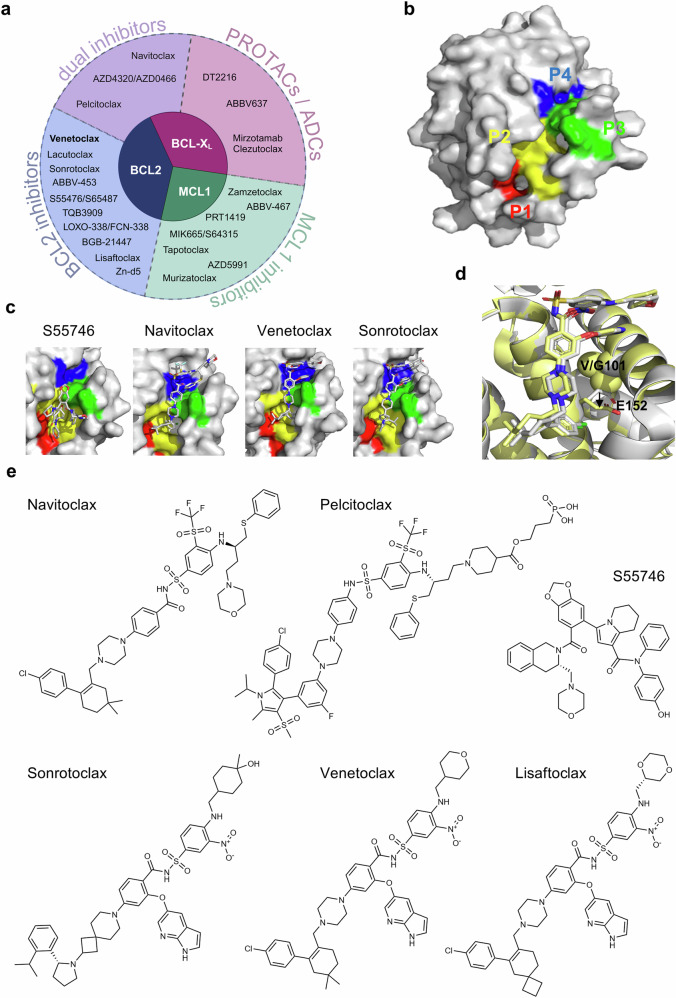
**Clinically developed BH3-mimetics**. **a** Overview of current clinically tested BH3-mimetics targeting BCL2, BCL-X_L_, MCL1 or multiple anti-apoptotic BCL2 proteins (created in Biorender). **b** Hydrophobic pockets P1, P2, P3 and P4 mapped onto the surface of the BCL2 structure (PDB-id: 1G5M^31^). **c** Zoom into SS55746, navitoclax, venetoclax or sonrotoclax bound to BCL2 (PDB-id: 6GL8,^336^ PDB-id: 4LVT,^32^ PDB-id: 6O0K,^320^ PDB-id: 8HOG). **d** Overlay of venetoclax bound to BCL2 (light grey) and BCL2-G101V (yellow). Mutation of glycine 101 (light grey spheres) to valine (yellow spheres) effects conformational change (indicated by black arrow) of the adjacent E152 (side chains as sticks in light grey and yellow, respectively), pushing it towards the chlorine (green) of venetoclax and slightly displacing it. (PDBids: 6O0K and 6O0L^320^). **e** 2D sketches of inhibitors shown in B-E, and pelcitoclax and lisaftoclax

The molecular function of BCL2 proteins in regulating cell survival highlights their potential as therapeutic targets for multiple diseases. Although the importance of BCL2 proteins is clearly demonstrated also in physiological tissues, this has not stopped the development and clinical assessment of multiple BH3-mimetics since the discovery of ABT-737 in 2005 (Fig. 5). While BCL2 proteins may also represent promising targets in autoimmune diseases (discussed below), the clinical development of BH3-mimetics has been centered on cancer, in particular lymphoid malignancies. The developed BH3-mimetics include bispecific inhibitors able to inhibit both BCL2 and BCL-X_L_, but also selective inhibitors binding only BCL2, BCL-X_L_ or MCL1 (Fig. 5a). Any clinical efficacy of BH3-mimetics will however depend on sufficiently large differences between the sensitivity of malignant and normal cells to BCL2 family inhibition, a situation comparable to regular chemotherapy.

Based on their mechanism of action, all true BH3-mimetics induce intrinsic apoptosis dependent on BAX and BAK, and therefore their effects should be inhibited in BAX/BAK-deficient cells.^275^ Selectivity of BH3-mimetics should also be confirmed by the high sensitivity of cell types that are known to be dependent on the targeted anti-apoptotic BCL2 protein for survival, e.g. CLL cells for BCL2 inhibitors^276^ and platelets for BCL-X_L_ inhibitors.^277,278^ This sensitivity profile highlights that BH3-mimetics may have profound on-target effects on non-malignant cells as also indicated by the phenotype of gene deletions in embryonic development.

#### The small molecule BH3-mimetics navitoclax and venetoclax

ABT-737 and Navitoclax: Due to its dysregulated expression in some hematological malignancies, BCL2 is a promising therapeutic target despite its broad expression in normal cells. The first potent and specific BH3-mimetic, ABT-737, targeted not only BCL2 but also BCL-X_L_ and BCL-w due to the structural similarities of their hydrophobic grooves. It binds to the hydrophobic pockets P2 and P4 of the anti-apoptotic proteins, displacing previously bound pro-apoptotic proteins like BIM or BAX (Fig. 5b and c). Navitoclax is an effective single-agent treatment for some B cell malignancies and non-small cell lung cancer cell and xenograft models with dependence on either BCL2 or BCL-X_L_ and low MCL1 expression.^27^ Single-agent treatment and combinations with immuno/chemotherapy phase I and II trials in lymphoid malignancies, particularly CLL, showed promising results.^279–281^ However, neutropenia and thrombocytopenia were major dose-limiting toxicities, the latter due to on-target effects of BCL-X_L_ inhibition.^277,279,282^ Consequently, many trials involving single-agent navitoclax have been completed/withdrawn. Current efforts are directed to combination regimes in Non-Hodgkin Lymphoma (NHL), myeloid neoplasms, ovarian cancer, triple-negative breast cancer, and non-squamous non-small cell lung carcinoma. Of particular note, two phase III trials (NCT04472598, NCT04468984) are currently investigating the combination of navitoclax with the JAK1/2 inhibitor, ruxolitinib, for the treatment of primary and relapsed/resistant (R/R) myelofibrosis, a form of myelodysplasia often characterized by increased platelet numbers and constitutive JAK/STAT activation.^283^ These trials followed a promising phase II trial.^284,285^ Early data from TRANSFORM-1 reported that the rate of spleen volume reduction was twice as high in patients who received navitoclax and ruxolitinib compared to placebo and ruxolitinib. Adverse effects of thrombocytopenia and neutropenia were commonly reported but manageable with navitoclax dose modification or interruption. Nonetheless, nearly one-third of patients discontinued treatment.^286^

Venetoclax: Navitoclax and related structures were reverse engineered through systematic removal or replacement of key binding elements to produce the first BCL2 specific inhibitor, venetoclax (ABT-199).^32^ The compound effectively kills various BCL2-dependent lymphoid cells and xenograft models in a BAK/BAX dependent manner, whilst sparing platelets.^32,287^ As of June 2024, venetoclax is investigated in 448 active clinical trials registered at clinicaltrials.gov, and among these are 40 phase III trials. In CLL, venetoclax is a transformative medicine. Clinically, nearly irrespective of their underlying genetic defects, all cases of CLL are exquisitely sensitive to BCL2 inhibition, usually with a low nanomolar EC50 in vitro. Consequently, venetoclax received several FDA breakthrough designations for the treatment of CLL following the success of several phase I and II studies.^34,35,288,289^ In May 2019, the FDA approved the use of venetoclax in combination with the CD20 antibody, obinutuzumab, as a chemotherapy free regimen for untreated CLL due the results of a phase III trial (CLL14) which found that venetoclax-obinutuzumab was associated with longer progression-free survival (PFS) than chlorambucil-obinutuzumab (88.2% vs 64.1% at 24 months).^290^ The 5-year follow up of this study reported patients treated with venetoclax-obinutuzumab continued to experience improved PFS (62.6% vs 27% in the chlorambucil-obinutuzumab arm) and higher rates of undetectable MRD.^291^ Early trials indicated a high risk of tumor lysis syndrome in the presence of bulk disease. Ramp-up dosage regimens and prophylaxis with hydration and rasburicase for high-risk patients were implemented which have greatly reduced the incidence and severity of this life-threatening complication^34^ and in most instances venetoclax can now be safely administered without hospital admission.

Additionally, full FDA approval was granted in 2020 for venetoclax in combination with hypomethylating agents for newly diagnosed AML patients older than 75 years who are not eligible for chemotherapy, based on the superior overall survival in the VIALE trial.^292,293^ Mechanistically, hypomethylating agents may reduce the expression of MCL1 while simultaneously increasing the expression of the BH3-only protein NOXA, which provides a rational molecular basis for the observed synergy with venetoclax.^294,295^ Additionally, increased T cell cytotoxicity has been reported following venetoclax treatment due to increased ROS formation and simultaneous activation of the cGAS/STING pathway activity. A more detailed understanding of the essential molecular mechanisms may pave the way for further refined combination treatments.^296,297^

In contrast, in FL and DLBCL, venetoclax monotherapy has been less successful, with reported response rates of only 38% and 18% respectively.^298^ Many FL and DLBCL cells express high levels of BCL2 due to either chromosomal translocation or amplification, and therefore such inherent resistance to BCL2 inhibition was unanticipated. Nonetheless, case reports of good responses after single-agent venetoclax in R/R DLBCL^299^ provide evidence for a subgroup of DLBCL with functional dependence on BCL2. Collectively, these data indicate that in DLBCL, unlike CLL, there is heterogenous dependence on different anti-apoptotic proteins, which is also supported by multiple preclinical studies.^300,301^ The disparity between anti-apoptotic protein expression and functional dependence is not exclusive to DLBCL and is therefore a poor biomarker to predict responses to BH3-mimetics.

Consequently, in DLBCL and other diseases, rational use of specific BH3-mimetics mandates robust identification of predictive biomarkers and this is now an area of intense research.^300–303^ Co-expression of alternative anti-apoptotic proteins, protein interactions and accessibility and phosphorylation status of BCL2 all likely contribute to venetoclax resistance in cases of high BCL2 expression. Additionally, in some instances, secondary genetic events in t(14;18) DLBCL may result in disruption of the *BCL2* gene resulting in loss of BCL2 protein expression.^304^ However, high expression of *BCL2* mRNA^305^ and BCL2 protein^306^ have been associated with poor outcomes in DLBCL; BCL2 “super-expressing” DLBCL may be associated with particularly poor outcomes.^306^ This has led to the empirical addition of venetoclax to various immuno-chemotherapy backbones in phase I/II clinical trials. Venetoclax in combination with immunochemotherapy (R-CHOP) in the CAVALLI phase II study indicated improved outcomes specifically in BCL2 highly expressing cases of DLBCL.^307^ More recently, the combination with the CD79B antibody drug conjugate (ADC), polatuzumab vedotin (NCT02611323) may be of interest since the ADC may cause downregulation of MCL1 *via* proteasomal degradation.^308^ Although the initial clinical results may look promising, in the absence of robust biomarkers associated with sensitivity to venetoclax, toxicities, both clinical and financial, have to be carefully weighed against perceived benefits.

In R/R multiple myeloma (MM), the chromosomal translocation t(11;14)(q13;q32.3) resulting in deregulated expression of Cyclin D1 has been suggested as a biomarker for venetoclax sensitivity.^309^ This subgroup of disease is associated with a B cell-specific transcriptional program resulting in upregulation of B cell genes such as MS4A1/CD20.^310^ Despite impressive results of single-agent treatment in R/R MM, adoption of venetoclax into combination regimens has been slowed by significant toxicities and deaths associated with increased infection rates in venetoclax-containing regimens.^311^ Although appearing in several treatment guidelines, venetoclax is not yet approved for use in MM.

Following the clinical success of venetoclax in the treatment of adult hematological malignancies, its use is currently also being evaluated in pediatric patients with leukemia, including AML and ALL. A phase I/II study of venetoclax in combination with chemotherapy shows promising response rates and manageable toxicities in pediatric AML patients.^312,313^ The combination of venetoclax with low-dose navitoclax and chemotherapy (NCT03181126) showed promising efficacy in ALL patients, and a safe dose of venetoclax could be identified for children.^314^ Of note, in pediatric T-ALL patients with a dismal prognosis, a beneficial effect of venetoclax was observed in R/R patients, highlighting the potential of venetoclax also in the treatment of childhood cancer (NCT03181126). However, as a note of caution it should be considered that developing neurons show increased expression of pro-apoptotic BCL2 proteins associated with higher apoptotic priming of neurons in children, indicating an important role of the BCL2 family proteins during brain development and neuronal differentiation.^142,315^ Therefore, the potential effects of venetoclax or similar drugs on the brain and neuronal development of children will need to be carefully assessed in long-term follow-up studies and may have to be compared to the serious long-term effects of intensive chemotherapy in children.

Resistance to venetoclax often emerges over prolonged treatment times and represents an important clinical challenge.^316,317^ Several acquired somatic *BCL2* mutations have been identified at the point of progression, the most prevalent being either G101V or D103Y.^318,319^ G101 is adjacent to but not directly a part of P4, and the P4 pocket was unaffected by mutations of this residue. Instead, the G101V mutation causes a rotamer change of E152 within the P2 pocket. This ‘knock-on’ effect reduces the affinity of BCL2 for venetoclax 180-fold, whereas the affinity for BH3-only proteins is largely unaffected (Fig. 5d).^320^ However, *BCL2* mutations are not identified in all CLL patients who relapse. Furthermore, in those that display mutated *BCL2*, the variant allele frequency is usually low, suggesting that there are multiple mechanisms that contribute to venetoclax resistance. Sub-clonal upregulation of alternative BCL2 family members, BCL-X_L_ and MCL1, have also been identified.^318,321^ This has been associated with copy number gains or increased NF-κB signaling which drives protein expression.^321,322^ Additionally, acquired variants of the effector BAX or loss of BAX protein have been reported in the myeloid compartment of venetoclax-resistant AML and CLL patients.^323,324^

**Fig. 6:**
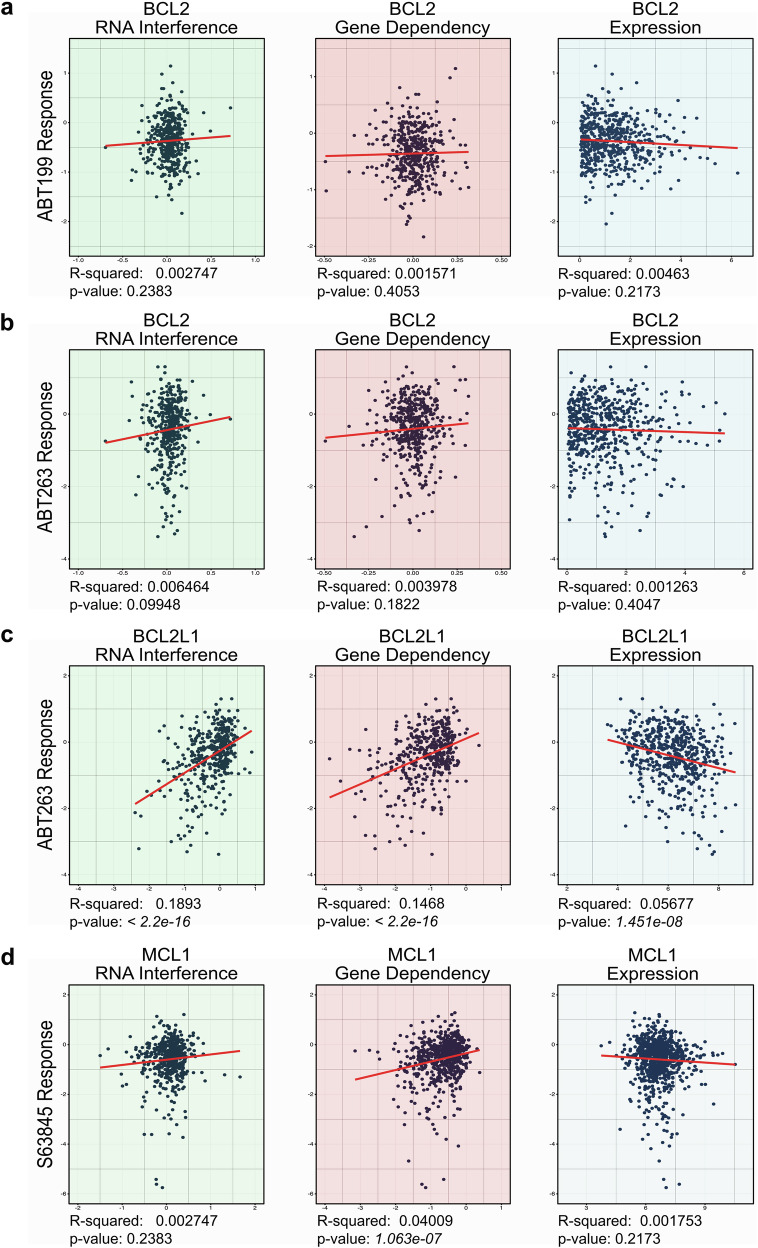
**Prediction of BH3-mimetic responses**. Linear regression analyses between cancer cell line response to BH3-mimetics from the PRISM Drug Repurposing Library and BCL2 RNA interference (green), BCL2 dependence score (pink) and BCL2 gene expression (blue). **a** Linear regression analyses between response to ABT-199 (venetoclax) and BCL2 (myeloid and lymphoid malignancies were not included in PRISM repurposing screening for ABT-199). **b** Linear regression analyses between response to ABT-263 (navitoclax) and BCL2. **c** Linear regression analyses between response to ABT-263 (navitoclax) and BCL2L1/BCL-X_L_. **d** Linear regression analyses between response to S63845 and MCL1. The most significant correlation is a negative one between *BCL2L1* expression and navitoclax response (**c**), however the R-squared value is only 0.06

Despite their high selectivity and binding affinity for the targeted anti-apoptotic BCL2 proteins, off-target effects of venetoclax have been described.^325^ A recent study described venetoclax as immunometabolic regulator in a subset of Natural Killer cells, an effect that was observed mainly independently of BCL2 expression.^326^ This may also speak to the non-canonical functions of the BCL2 family, some of which may not be inhibited by BH3-mimetics. The autophagy promoter, beclin 1 (*BECN1*), contains a BH3 domain which can be inhibited via interactions with BCL2 and BCLX_L_. In line with this, BH3-mimetics such as ABT-737 have been proposed to induce autophagy.^327^ Additionally, the BH4 domain of BCL2 has been implicated in autophagy regulation. It has been reported to interact with GABA Receptor Associated Protein (GABARAP), a molecule involved in autophagosome biogenesis^328^ and overexpression of BCL2 lacking its BH4 domain commits melanoma cells to autophagy.^329^ Interactions of the BH4 domains of BCL2 and BCL-X_L_ have also been linked to roles in proliferation, differentiation, DNA repair, cell migration and tumor progression.^330^ These interactions would not be hindered by BH3-mimetics. Other reported non-canonical functions of the anti-apoptotic BCL2 family members include regulation of senescence, inflammation, metabolism, mitochondrial morphology, and calcium homeostasis.^331,332^ With so many roles of these proteins above and beyond suppression of apoptosis, it is perhaps unsurprising that in a large screen across different cancer types there was a poor correlation between *BCL2* family RNA expression and BH3-mimetic sensitivity (Fig. 6). Some of this disparity may be due to this correlation being based on RNA expression rather than protein expression. However, target protein expression has been defined as a poor biomarker of BH3-mimetic sensitivity in multiple cancer models.^301,333–335^ In a pan-cancer analysis, the gene knockout effects do not resemble the response to venetoclax or navitoclax, and only an association between navitoclax response and a *BCL2L1* gene dependency is observed (Fig. 6). Taken together, these studies highlight that non-canonical functions of BCL2 proteins as well as unanticipated off-target effects of venetoclax or navitoclax need to be considered in the assessment of clinical responses.

#### Other BCL2 targeting BH3-mimetics

As frequently happens in the pharmaceutical industry, the success of venetoclax has spawned a plethora of structurally related molecules, some of which differ only slightly from venetoclax (Fig. 5e). In total, 11 selective BCL2 inhibitors have entered clinical trial; development of some has already been discontinued (Table 1).

**Table 1:** BH3-mimetics in clinical development

Name	Target	Status	Original Ref
Venetoclax / ABT-199	BCL2	FDA approved	^32^
Sonrotoclax / BGB11417	BCL2	Phase III	^338^
Lisaftoclax / APG-2575	BCL2	Phase III	^527^
ABBV-453	BCL2	Phase I	unpublished
ABBV-623	BCL2	Terminated	unpublished
S55746 / BCL201	BCL2	Phase I completed	^336^
S65487 / VOB560	BCL2	Phase I (halted)	^337^
FCN-338 / LOXO-338	BCL2	Phase I	^346^
BGB21447	BCL2	Phase I	unpublished
ZN-d5	BCL2	Phase I/II	^344^
Lacutoclax / LP-108	BCL2	Phase I/II	^350^
TQB3909	BCL2	Phase I/II	unpublished
Navitoclax / ABT-263	BCL2/BCL-X_L_	Phase III	^27^
AZD4320 and AZD0466	BCL2/BCL-X_L_	Terminated	^438^
Pelcitoclax / APG-1252	BCL2/BCL-X_L_	Phase I	^365^
Mirzotamab Clezutoclax / ABBV-155 (ADC)	BCL-X_L_	Phase I (halted)	^441,442^
ABBV-637 (ADC)	BCL-X_L_	Phase I (halted)	^443^
MIK665 / S64315	MCL1	Phase I (halted)	^370^
AZD5991	MCL1	Phase I	^539^
Tapotoclax / AMG176	MCL1	Phase I (halted)	^373^
Murizatoclax / AMG397	MCL1	Terminated	^375^
Zamzetoclax/ GS9716	MCL1	Phase I	^378^
ABBV-467	MCL1	Terminated	^371^
PRT1419	MCL1	Terminated	^377^

ABBV-543 and ABBV-623: In addition to venetoclax, AbbVie (www.abbvie.com) developed two further BCL2 inhibitors. ABBV-453 is a BCL2 inhibitor in a phase I trial for patients with R/R t(11;14) positive and/or BCL2 high-status MM (NCT05308654). Another trial investigating the efficacy of ABBV-453 in R/R CLL/SLL patients who have had at least two prior therapies (NCT06291220) has been recruiting since August 2024. The other compound, ABBV-623 has been tested in a clinical trial (NCT04804254) but its development has now been discontinued. No chemical structures or biological data have been published for either compound.

S55746 and S65487: S55746 (BCL201) is a BCL2-specific inhibitor developed by Servier (https://servier.com) using structure-based drug design. The compound activated apoptosis in CLL and MCL patient samples and induced tumor regression in xenograft models at a comparable level to venetoclax.^336^ In marked contrast to venetoclax, S55746 binds *via* a different binding mode and mainly occupies pockets P1, P2 and P3 but not P4 of BCL2 (Fig. 5c). Despite alternative binding modalities, S55746 binds to G101V mutant BCL2 with 100-fold lower affinity compared to wildtype BCL2.^320^ Two clinical trials were completed; firstly, to assess the safety and tolerability of S55746 in CLL and NHL (NCT02920697), and secondly, to assess the combination of S55746 and the PI3K inhibitor, idelalisib, in FL and MCL (NCT02603445). Despite having been completed six years ago, no results from these trials are available. S65487 (VOB560), a prodrug of S55746, induced apoptosis in BCL2-dependent hematological cell lines and displayed synergy when combined with MCL1 inhibitors in AML patient-derived xenografts.^337^ Additionally, S65487 was potent in pre-clinical models resistant to venetoclax, including those that harbored *BCL2* mutations.^337^ S65487 is being assessed clinically in combination with a MCL1 inhibitor for the treatment of R/R AML, MM or NHL (NCT04702425) or in combination with azacitidine in patients with untreated AML (NCT04742101), but both studies are currently not recruiting.

Sonrotoclax and BGB-21447: Beigene (www.beigene.com) produced a series of BCL2 inhibitors through scaffold screening and structure-based drug design leading to the development of sonrotoclax (BGB-11417), which binds to BCL2 with higher affinity than venetoclax due to a different binding mode within the P2 pocket^338^ (Fig. 5c). Stronger binding was attributed to van der Waals forces, additional hydrophobic interactions, and water-bridged hydrogen bonds not present in BCL2:venetoclax interaction. Moreover, it was more potent in BCL2-dependent hematological tumor cells and xenograft models compared to venetoclax. Interestingly, due to its unique binding mode, sonrotoclax can inhibit the G101V BCL2 variant and kill mutant cells.^338,339^ There are currently seven active phase I and II clinical trials involving sonrotoclax monotherapy for the treatment of mature B cell malignancies, R/R CLL and SLL, R/R MCL, myeloid malignancies and Waldenström Macroglobulinemia. There are an additional two studies (phase II and III) that combine sonrotoclax and the BTK inhibitor, zanubrutinib, in participants with untreated CLL. Early clinical trial data were promising, indicating that sonrotoclax was well tolerated and achieved good responses both as monotherapy^340^ and in combination with zanubrutinib in R/R and treatment naïve CLL/SLL.^341,342^ BGB-21447 is another BCL2 inhibitor, for which there are no published data, however there is a phase I clinical trial in mature B cell malignancies underway (NCT05828589).

Lisaftoclax: The development of lisaftoclax (APG2575) by Ascentage (www.ascentage.com) marked another selective BCL2 inhibitor that is orally active and potently inhibits BCL2.^343^ Its overall structure is based on venetoclax but displays key structural differences (Fig. 5e) leading to faster cellular uptake and slightly higher cell death induction in CLL cells. Data from the phase I clinical trial in CLL patients indicated promising results with rapid clinical responses and an acceptable safety profile.^344^ Similar to sonrotoclax, lisaftoclax may potentially maintain activity in AML cells with acquired resistance to venetoclax when applied in combination with agents that upregulate BAX or downregulate MCL1.^345^ Following promising early clinical data, lisaftoclax is currently being investigated in a phase III trial in China for the combination with azacitidine in AML patients (NCT06389292) and in a global phase III trial in combination with BTK inhibitors in CLL patients (NCT06104566).

FCN-338/LOXO-338: FCN-338 is a selective BCL2 inhibitor developed by Fochon Pharmaceutical (https://fochonpharma.com) which displayed nanomolar potency against a range of BCL2-dependent FL, DLBCL, AML and ALL cell lines in vitro.^346,347^ Dose-dependent tumor growth inhibition was observed in FL, AML, and ALL xenograft models without significant weight loss. In October 2020, LOXO Oncology at Lilly (https://www.loxooncology.com) obtained the rights to develop and commercialize the drug under the name LOXO-338. Initial results from the first-in-human phase I study with advanced hematological malignancies (NCT05024045) showed that LOXO-338 was well tolerated and efficacy was observed.^348^ A second part of this trial was to evaluate LOXO-338 plus the BTK inhibitor, pirtobrutinib (LOXO-305), which was effective in vitro.^349^ However, in November 2022, Lilly discontinued the development of this molecule. A phase I trial by Fochon Pharmaceuticals is still recruiting patients with R/R CLL/SLL in China to investigate FCN-338 (NCT04682808).

Zn-d5: Zn-d5 is a selective BCL2 inhibitor developed by Zentalis Pharmaceuticals (https://www.zentalis.com) which reportedly demonstrates significant single-agent and combination anti-tumor activity in xenograft models of NHL, AML, and other hematological and solid tumor types.^344^ It has been under clinical assessment for R/R NHL, AML and light chain amyloidosis. Additionally, the combination of Zn-d5 and the WEE1 inhibitor azenosertib was being evaluated in a phase I/II trial for AML (NCT05682170), which has been terminated.

LP-108: Newave Pharmaceuticals (http://www.newavepharma.com) is clinically developing a BCL2 inhibitor named LP-108 (lacutoclax). Initial data from a phase I clinical trial in R/R CLL and NHL (NCT04356846) indicate that the drug is well tolerated with an overall response rate (ORR) of 53.8%.^350^ For R/R CLL/SLL the ORR was 75%. Monotherapy of LP-108 and combination with azacitidine is also under assessment for the treatment of R/R AML, MDS, and chronic myelomonocytic leukemia. Early data shows the combination has an advantageous ORR and PFS compared to single-agent LP-108.^351^ The Newave pipeline also indicates an additional BCL2 inhibitor, NW-4-1191, still in the discovery stage.

TQB3909: In China, the Chia Tai Tianqing Pharmaceutical Group (www.cttq.com) are the sponsors of several phase I trials evaluating the efficacy of the BCL2 inhibitor, TQB3909, in patients with R/R NHL, AML, MCL, plasmacytoma, myelofibrosis, MDS or HR-positive and HER2 negative advanced or metastatic breast cancer. First results presented at the European Society for Medical Oncology in 2024 indicate clinical activity particularly in the CLL patient population with an acceptable safety profile.^352^

Taken together, several selective BCL2 targeting BH3-mimetics are emerging clinically that may compete with venetoclax for the treatment of hematological malignancies. Whether any will outperform venetoclax remains to be seen; in CLL, venetoclax, particularly in combination with BTK inhibitors and obinutuzumab has set a very high bar. Determination of clinically significant differences will mandate long-term, head-to-head phase III clinical trials with careful assessment of toxicities as well as efficacies in terms of minimal residual disease assessments. Additionally, associated treatment cost differentials may play a major role in determining which inhibitor will be used as standard of care. With sonrotoclax and lisaftoclax now in phase III clinical trials, further clinical results are eagerly awaited.

#### Other BCL-X_L_ and BCL2/BCL-X_L_ dual inhibitor BH3-mimetics

Since BCL-X_L_ is widely overexpressed and an essential gene in multiple solid tumors (Figs. 3 and 4), compounds that selectively target BCL-X_L_ may have the potential to translate promising results from hematological malignancies into the field of solid tumors dependent on BCL-X_L_ for survival. AbbVie introduced the compound WEHI-539 in 2013 as the first highly selective inhibitor of BCL-X_L_.^353^ Although several preclinical studies showed effective antitumor activity either as a monotherapy or as a combination treatment for various entities, WEHI-539 was never clinically tested.^354–356^ AbbVie presented two further BCL-X_L_ inhibitors with improved selectivity over the next few years, A1155463 and A1331852, which have been in preclinical research since 2014 and 2015 respectively.^357,358^

In preclinical research, A1155463 has been shown to be effective at low nanomolar concentrations against colorectal cancer cells with high expression of BCL-X_L_, as well as other solid malignancies.^359,360^ A1331852 similarly has been shown to induce apoptosis across a variety of entities, but as well as A1155463, was used mainly as a tool compound to investigate the importance of singular anti-apoptotic BCL2 family members. Of note, a third compound named A1293102 was presented in 2021 by AbbVie, however, no further preclinical studies have been conducted so far.^361^ None of these compounds have entered clinical assessment.

Following the dual BCL2/BCL-X_L_ inhibitors ABT-737 and navitoclax, several other comparable approaches using dual inhibitors are being investigated with the goal of reducing dose-limiting side effects while maintaining the efficacy and wide-range applicability. APG-1252 (pelcitoclax) developed by Ascentage is a phosphate prodrug that is converted to APG-1252-M1 after reaching its target cells.^362^ It has higher affinity for BCL2 and BCL-X_L_ compared to navitoclax and lower permeability in platelets. APG-1252 at submicromolar concentrations was shown to achieve strong in vitro effects in gastric cancer cells, as well as colorectal and non-small cell lung cancer cells.^362–364^ A phase I clinical trial investigated APG-1252 safety in a cohort of patients with locally advanced or metastatic solid tumors reporting good tolerance and antitumor effects.^365^ Four studies investigating APG-1252 for the treatment of NHL or solid tumors, alone and in combination with other precision medicines, are currently recruiting (NCT05186012, NCT04001777, NCT04893759, NCT05691504).

#### MCL1-selective BH3-mimetics

MCL1 amplification is associated with higher grade malignancy and poor prognosis in many malignancies.^366–368^ Structurally, in contrast to BCL2 and BCL-X_L_, MCL1 has only one central hydrophobic helix surrounded by six helices which are less densely packed giving rise to a wider hydrophobic binding pocket.^29^ This has made the development of inhibitors more challenging. Nonetheless, several MCL1-specific BH3-mimetics have been developed by different pharmaceutical companies or academic institutions. These compounds target one or more of the four pockets within the hydrophobic groove of MCL1, and several MCL1 inhibitors have entered clinical studies as single agent or in combination with other drugs. However, all but one clinical trials have been halted over safety concerns and none have yet received approval for clinical usage (Table 1).

S63845 and S64315: S63845 has been shown to be efficient in killing cells from various hematologic malignancies and displays potent anti-tumor activity as a single agent in numerous malignancies in vivo.^369^ Its derivative MIK665/S64315^370^ was tested in several phase I dose escalation studies for the treatment of R/R lymphoma and MM (NCT02992483), AML, and MDS (NCT02979366). In addition, a dose-escalation trial of S64315 in combination with venetoclax (NCT03672695) was conducted and a phase I/II trial (NCT04629443) evaluating the combination of S64315 and azacitidine in AML patients has been completed but formal results have not been presented. According to data posted on the clinical trials website (https://clinicaltrials.gov/study/NCT04629443?tab=results accessed 24JUN2024) 17 patients were entered, but none completed the study due either to progressive disease (8 patients) or adverse events (4 patients); there were four fatal adverse events. A major limitation of this molecule is the necessity for intravenous administration at three times per week. With this dosing schedule, efficacy may be limited given the short half-life of MCL1 protein.

ABBV-467: ABBV-467, a very potent and selective macrocyclic MCL1 inhibitor with a short half-life in humans, was tested for safety and tolerability in adults with R/R MM (NCT04178902). While treatment with ABBV-467 achieved some disease control, it also caused elevated cardiac troponin levels in the plasma in some patients, prompting the study’s termination, despite the absence of other cardiac findings.^371^ The effect of MCL1 inhibitors on cardiomyocytes was also predicted by genetic studies showing cardiomyopathy and heart failure in cardiomyocyte-specific MCL1 knockout mice,^372^ indicating on-target toxicity of the MCL1 inhibitors.

AMG176 and AMG397: AMG176 (tapotoclax) is a potent, specific, and orally available MCL1 inhibitor developed by Amgen (www.amgen.com).^373^ Following an initial safety study (NCT02675452), AMG176 was investigated in combination with venetoclax in patients with R/R hematologic malignancies (NCT03797261), which has been suspended, and in combination with azacitidine in patients with MDS/CML (NCT05209152), which has been completed. However, no results from either study have been posted, despite the former study completing in 2019. Improvement of AMG176 resulted in AMG397 (murizatoclax)^374^ which has high affinity for MCL1 and selectively competes with pro-apoptotic BCL2 family members for the binding to the BH3-binding groove of MCL1. AMG397 effectively disrupted the interaction between MCL1 and BIM, increased caspase-3/7 activity and reduced cell viability. Cell lines from hematologic malignancies were highly sensitive to AMG397, and in vivo oral treatment led to significant tumor regressions in xenograft mice.^375^ A phase I dose-escalation study of AMG397 in MM, NHL or AML was undertaken (NCT03465540) but terminated due to cardiac toxicity. As with S63415, preliminary data available on the clinical trials website indicate significant toxicities without major responses (https://clinicaltrials.gov/study/NCT03465540).

AZD5991: AZD5991 is another highly selective macrocyclic MCL1 inhibitor developed by Astrazeneca (www.astrazeneca.com). Clinical studies using it either alone or in combination with venetoclax in patients with R/R hematologic malignancies (NCT03218683) were conducted and stopped, most likely due to toxicities. A trial of biomarker-based therapy for AML initially incorporated AZD5991 (NCT03013998), but the arm containing AZD5991 has been closed.

PRT1419: PRT1419 is a selective inhibitor of MCL1 developed by Prelude Therapeutics (https://preludetx.com) that has demonstrated preclinical efficacy in hematologic malignancies and solid tumors. A phase I dose expansion study of PRT1419 in R/R hematologic malignancies (NCT04543305) was carried out to evaluate the dosing schedule, maximum tolerated dose, and/or the optimal biological dose. A separate study (NCT05107856) tested PRT1419 as a once weekly intravenous infusion in patients with hematologic malignancies, using it as monotherapy or in combination with azacitidine or venetoclax in R/R myeloid or B cell malignancies. Given that MCL1 overexpression is a modulator of venetoclax resistance^317^ and azacitidine downregulates MCL1,^376^ PRT1419 may provide additional or synergistic advantages to azacitidine or venetoclax in treatment-resistant myeloid or B cell malignancies. However, this study was terminated in January 2024; no results have thus far been posted. Furthermore, MCL1 inhibition with PRT1419 in patients with advanced solid tumors (R/R breast and lung cancer, sarcoma and melanoma) (NCT04837677) indicated satisfactory safety and tolerability, with no cardiac toxicity reported. BAX and caspase 3 activation were observed, suggesting potentially successful MCL1 inhibition, however no significant efficacy was observed.^377^ Whether PRT1419 is being further developed by the company is not clear; the molecule is not currently listed on the company website (accessed 24JUN2024).

GS9716: GS9716 (zamzetoclax) developed by Gilead (www.gilead.com) is a potent and specific small molecule inhibitor that binds directly to MCL1. In vivo and in vitro studies have demonstrated a strong single-agent effect as well as synergy with other anti-cancer therapies.^378^ GS9716 is currently undergoing a phase I clinical trial in patients with advanced solid tumors to assess its safety, tolerability, and pharmacokinetics as monotherapy and in combination with anticancer medicines (NCT05006794). This makes GS9716 the only MCL1 inhibitor currently being given to patients, but since its original presentation at AACR in 2022,^378^ no scientific update has been provided.

Additional putative inhibitors of MCL1 are currently emerging, which have not yet entered clinical testing. NA1-115-7 is a compound isolated from the Winteraceae plant *Zygogynum pancheri* native to eastern and south eastern New Caledonia.^379^ The physicochemical properties and structural complexity of natural products make them particularly interesting for use in the treatment of cancer. NA1-115-7 binds to MCL1 in the BH3 binding groove and forms a covalent bond, making it the first covalent BH3-mimetic that targets MCL1. In lymphoma cells, NA1-115-7 stabilized MCL1, disrupted the BAK/MCL1 interaction, and rapidly induced apoptosis in a BAK- and BAX-mediated process.^380^ Moreover, NA1-115-7 induced tumor cell death while demonstrating no toxicity to cardiomyocytes or normal blood cells.^380^ Like many other natural compounds, it has a lipophilic structure, and its susceptibility to acidic environments may impede its oral administration and, consequently, its clinical development. A NA1-115–7-loaded nanoemulsion was created to address these issues, which could potentially render it easier to evaluate in vivo.^381^

In summary, despite the successful development of potent and selective MCL1 inhibitors, their toxicities observed in patients have so far precluded clinical development. These toxicities highlight the need to develop novel strategies to safely deliver MCL1 inhibitors to the intended target cells while sparing healthy tissues.

### Novel approaches to target BCL2 proteins

#### Cyclic peptides

As an alternative to small BH3-mimetics, also cyclic or stapled peptides may be employed to target BCL2 proteins. These peptides are usually based on the BH3 domain and modified to ensure high affinity binding to the targeted anti-apoptotic BCL2 protein as well as improved stability. Pioneering work by the Korsmeyer lab using hydrocarbon stapling resulted in the generation of the first stabilized alpha-helix of BCL2 domains (SAHBs) displaying high binding affinity to BCL2 or BAX and cell permeability.^382,383^ A stapled-modified BIM BH3 peptide has been shown to reactivate apoptosis and overcome resistance by simultaneously inhibiting multiple BCL2 proteins.^384,385^ Modification of the BH3 domain sequence and inclusion of non-natural amino acids and acrylamide moieties resulted in the development of SAHBs selectively targeting BCL2A1, highlighting the potential of this approach in targeting BCL2 proteins that were so far not amenable for the development of selective BH3-mimetics.^386–388^ Recent research has also demonstrated that SAHBs may be designed to inhibit specific functions of BCL2 proteins, e.g. the interactions with IP_3_Rs, to dissect the functions of BCL2 proteins at different subcellular compartments.^389^ However, so far these approaches have been limited to preclinical tool compounds and have not yet been tested clinically. To the best of our knowledge, the only stapled peptide to enter clinical trials (NCT02909972, NCT03654716)^390^ was the MDM2 inhibitor ALRN-6924 (sulanemadlin)^391^ and development of this compound has now been terminated, indicating that the translational value of stapled peptides beyond their use as tool compounds remains to be proven.

#### BH4 domain-targeting of BCL2

As anti-apoptotic BCL2 also operates as an inhibitor of IP_3_R channels, strategies to disrupt IP_3_R/BCL2 complexes in cancer cells have been investigated. Most strategies have focused on IP_3_R-derived peptides that comprise the BH4 domain-binding site of BCL2. As such, these tools serve as a decoy for BCL2 and strip IP_3_Rs from BCL2. A cell-permeable stabilized version of this peptide, BCL2/IP_3_R disrupter-2 (BIRD-2), has been applied in different BCL2-dependent cancer cell types. BIRD-2 application by itself, in the absence of other pro-apoptotic triggers, was sufficient to kill CLL and DLBCL cells by triggering excessive, pro-apoptotic Ca^2+^ release.^392,393^ Further mechanistic studies revealed that BIRD-2 sensitivity in B cell lymphoma cells was particularly due to the combination of two factors: first, high levels of IP_3_R2, the IP_3_R isoform with the highest sensitivity to IP_3_,^393^, and second, constitutive basal IP_3_ signaling downstream of the tonically activated B cell receptor.^394^ Moreover, a reciprocal sensitivity between BIRD-2 and venetoclax has been reported across a panel of DLBCL cell lines.^395^ However, BIRD-2 enhances the sensitivity of DLBCL cells to venetoclax by evoking Ca^2+^-dependent upregulation of BIM.^395^ The synergistic cell death effects between BIRD-2 and BH3-mimetics also have been exploited in several other cancer types including MM, FL and small cell lung cancer cells.^396,397^ In addition, BIRD-2 has been combined with chemotherapeutic agents, which enhanced the cell death response in ovarian cancer cells.^398^

Beyond peptides, also BH4 domain-targeting BCL2 inhibitors have emerged. BDA-366 was the first molecule reported to directly bind to the BH4 domain of BCL2 and thus to serve as a BH4 antagonist, capable of killing lung cancer and MM cells.^399,400^ The mechanism for this cell death effect was attributed to its ability to convert BCL2 into a pro-apoptotic protein by triggering the exposure of its BH3 domain, thereby resulting in BAX/BAK activation.^401^ As such, cancer cells with high levels of BCL2 were more susceptible to BDA-366. However, further work, at least in BCL2-dependent malignant B cells, indicated that BDA-366 evoked cell death independently of BCL2 protein levels, even potently killing cancer cells lacking substantial BCL2 protein levels.^402^ Furthermore, in vitro BAX pore-forming assays excluded that BDA-366 either directly or indirectly through BCL2 could activate BAX pore formation.^402^ Hence, the mechanism of action of BDA-366 in evoking cancer cell death requires further scrutiny.^403^ Furthermore, other BH4 domain-antagonizing small molecules have emerged such as CYD0281, which limited breast cancer growth^404^ and DC-B01, which kills lung cancer cells.^405^ DC-B01 has been reported to block the BCL2/cMYC complex, thereby suppressing the transcriptional activity of cMYC. Moreover, lung cancer cells in which BCL2 was knocked down became resistant to DC-B01, indicating an on-target effect of DC-B01. Yet, further work is needed to assess its effect in other BCL2-dependent cancer types. Finally, these peptides and small molecules have so far been used in pre-clinical settings, and thus further research is needed to determine whether a strategy targeting the BH4 domain may lead to novel therapeutics.

#### PROTACs

Therapeutic targeting of BCL-X_L_ in men resulted in the dose-limiting on-target toxicity of thrombocytopenia. Therefore, a lot of effort has been devoted to developing other strategies to target BCL-X_L_ without inducing platelet toxicity. One approach has been to develop BCL-X_L_ or BCL-X_L_ /BCL2 dual PROTACs (reviewed in^406^). PROTACs are advantageous in this setting as they promote proteasome-mediated degradation of the target protein through recruitment of E3 ubiquitinating ligases, allowing for tissue-specificity based on the expression of E3 ligases. Although there are around 600 E3 ligases in the human genome,^407^ most of the clinically advanced PROTACs are based on Von Hippel-Lindau (VHL) cullin-2, Cereblon (CRBN) cullin-4A RING, Mouse Doubleminute 2 homolog (MDM2) or Inhibitor of Apoptosis Proteins (IAP) as recruited E3 ligases.^408^ As VHL and CRBN ligases are only expressed at low levels in platelets,^409^ unwanted toxicity of BCL-X_L_ inhibition may be strongly reduced. An additional advantage of PROTACs over small molecule inhibitors lies in their ability to promote target degradation catalytically, enabling more effective target neutralization at lower drug exposure.^410^

In addition to the E3 ligase ligand, PROTACs consist of a so-called warhead, which is often a small molecule inhibitor against the protein of interest. Those functional groups are connected through a linker of variable length and properties that are designed to ensure that the linker does not disturb the binding properties of the warhead. This general toolbox consisting of warhead, linker, and E3 ligase ligand offers various combinations and design strategies, which can be seen in the overview of PROTACs in Table 2.

**Table 2:** BCL2 protein targeting PROTACs and SNIPERs

Name	Target	Clinical	Warhead	E3 ligase	Original Ref
DT2216	BCL-X_L_	Yes	ABT-263	VHL	^411^
XZ739	BCL2 (inh), BCL-X_L_	No	ABT-263	CRBN	^415^
SIAIS361034	BCL2 (inh), BCL-X_L_	No	ABT-263	CRBN	^417^
PZ15227	BCL-X_L_	No	ABT-263	CRBN	^540^
XZ424	BCL-X_L_	No	A1155463	CRBN	^419^
PZ703b	BCL2 (inh), BCL-X_L_	No	ABT-263	VHL	^421^
753B	BCL2/BCL-X_L_	No	ABT-263	VHL	^423^
WH244	BCL2/BCL-X_L_	No	ABT-263	VHL	^425^
C3	MCL1	No	Nap-1	CRBN	^427^
C5	BCL2/MCL1	No	Nap-1	CRBN	^427^
PROTAC-8a	BCL-X_L_	No	ABT-263	IAPs	^420^
NXD02	BCL-X_L_	No	Modified	VHL	^426^

The furthest advanced BCL-X_L_-targeting compound DT2216 developed by Dialectic Pharmaceuticals (www.dtsciences.com) is in clinical testing in a phase I trial in R/R malignancies, including solid and hematological cancers (NCT04886622). DT2216 is based on ABT-263 linked to a VHL recruiting ligand. Although ABT-263 has high binding affinity against BCL-X_L_ and BCL2, it only forms ternary complexes with BCL-X_L_ in living cells. In this ternary complex, VHL ligase can transfer ubiquitin to Lys87 of BCL-X_L_.^411^ The inability to degrade BCL2 might be caused by the absence of a stable ternary complex. The specific targeting of BCL-X_L_ by DT2216 has proven effective against BCL-X_L_-dependent cancers^412^ and it has been shown that DT2216 is also able to overcome BCL-X_L_-mediated gemcitabine resistance, without causing organ toxicity.^413^ Furthermore, breast, prostate, liver and colon cancer cell lines with acquired BCL-X_L_-mediated resistance against commonly used chemotherapeutic agents could be sensitized by DT2216 treatment.^411^ In vitro studies combining DT2216 with small molecule MCL1 inhibitors in small-cell lung cancer showed promising results in addressing the co-dependency of the cancer cells without induction of toxicity in healthy cells.^414^ Initial preclinical studies comparing the effects of DT2216 and ABT-263 side-by-side in platelets and leukemia cells indicate a highly improved therapeutic window for DT2216.^411^ DT2216 has received FDA fast-track designation for adult patients with R/R peripheral and cutaneous T cell lymphomas (https://prn.to/3O2PiGm), but no recent updates have been provided.

Three other PROTACs targeting BCL-X_L_ also through ABT-263 rely on pomalidomide as CRBN recruiting ligand. SIAIS361034, PZ15227 and XZ739 differ by their linker design, which can affect the ability to form stable ternary complexes and therefore the effectivity of the PROTAC to degrade the targeted protein. In PZ15227, ABT-263 is connected via a piperazine ring to the linker, similar to DT2216, whereas ABT-263 is tethered through a N-methylamino group to a PEG linker in XZ739. XZ739 showed improved potency compared to DT2216, induced apoptosis in T-ALL cell lines and displayed indications for senolytic activity,^415^ indicating on-target activity.^416^ While SIAIS361034 binds both BCL2 and BCL-X_L_, it only degrades BCL-X_L_ and has proven selective in hepatocellular carcinoma.^417,418^ XZ424 is also a CRBN-based PROTAC, but instead of the dual inhibitor ABT-263 it uses the selective BCL-X_L_ inhibitor A1155463. The PROTAC exhibited similar binding affinities to BCL-X_L_ as the small molecule inhibitor, but proteasome-mediated BCL-X_L_ degradation was not accompanied by platelet toxicity.^419^

To provide further options for cells with limited response to VHL or CRBN-based PROTACs, PROTAC-8a was developed using the IAP antagonist 1 bound to ABT-263 through a short alkane linker. BCL-X_L_ degradation was observed across different entities of solid cancers, whereby the sensitivity seemed to correlate with XIAP E3 ligase expression.^420^

In addition to the linker properties, the orientation of the warhead also largely influences the interaction with the targeted protein and thereby its degradation specificity. This becomes obvious in the dual-targeting BCL-X_L_/BCL2 PROTACs PZ703B, 753B, and WH244 which are also based on ABT-263 as a warhead, but with altered orientations. This enables a dual targeting mechanism where PZ703B degrades BCL-X_L_ and inhibits BCL2 function.^421^ 753B differs in the linker domain. Furthermore, both compounds exerted enhanced degradation potency compared to their respective stereoisomers, highlighting that in addition to the tethering position of the warhead, also the steric orientation impacts the ternary complex formation. Although 753A and B displayed similar binding activity against both BCL-X_L_ and BCL2, their binding was weaker than that of the parent inhibitor ABT-263. However, 753B showed single-agent potency in reducing both target proteins in AML models and senolytic activity through apoptosis induction.^422^ The capacity to also target BCL2 is given by the different ternary complex conformation compared to DT2216, allowing access to BCL2 surface lysines as ubiquitination sites.^423^ In small-cell lung cancer 753B was also more potent than DT2216 or ABT-263 in reducing cell viability and was well tolerated in mice.^424^ With PROTAC 753B as a starting point, the second-generation dual degrader WH244 was recently developed. By studying the ternary complex formation of VHL/753B and BCL2 or BCL-X_L_, it was possible to optimize the linker design and improve potency for both BCL-X_L_ and BCL2 degradation, resulting in a highly promising compound that exhibited enhanced activity in leukemia cells.^425^

A slightly different approach was recently presented by NeoXBiotech (www.neoxbio.com) in the design of its lead compound NXD02.^426^ In comparison to the navitoclax-based PROTAC DT2216, NXD02 was designed to have less potent binding for BCL-X_L_, to prevent an inhibitory effect while maintaining the ability to degrade BCL-X_L_, and thus establishing a favorable therapeutic window and preventing platelet toxicity.

So far, only few reports describe specific and potent MCL1 targeting PROTACs. Compound C3 is described to selectively degrade MCL1 while compound C5 from the same synthesis trials targets BCL2.^427^ Both compounds work via CRBN recruitment and are based on the dual BCL2/MCL-1 inhibitor Nap-1,^428^ showcasing that the linker design influences the properties of the PROTAC molecule, not only concerning solubility and membrane permeability, but also regarding specificity and degrading potency.

Targeting of BCL2 may also be possible using molecular glue degraders, and very recently several thalidomide-related compounds have been described that selectively target BCL2. These thalidomide analogs bind close to the BH1 domain of BCL2 to recruit CRBN, thus holding the potential to also target mutant BCL2 proteins emerging as a mechanism of venetoclax resistance.^429^

Additionally, efforts are made to further reduce undesirable off-target effects of PROTACs by the introduction of PROTAC prodrugs that are recruited to cancer cells through biomarker recognition. Here, the PROTAC function is activated in a biomarker-dependent fashion, which ensures the degradation of the targeted BCL2 protein selectively in the cancer cells.^430^ This approach highlights an entirely new strategy, in which PROTACs may be more selectively delivered to the intended cell types.

#### Nanoparticles and antibody drug conjugates

The very first clinically tested attempts to target BCL2 relied on antisense technology used in G3139 (oblimersen),^431,432^ and hence the ongoing clinical trials with BP1002 (NCT05190471 and NCT04072458) read a bit like a trip down memory lane. This liposomal BCL2-targeting antisense DNA molecule has been developed by Bio-Path (https://www.biopathholdings.com) using neutral charge lipids as carriers to improve stability and overcome toxicity of previous approaches. An optimized siRNA-based approach to target MCL1 has recently been described that may also achieve better plasma stability due to increased albumin binding and that may be able to reduce MCL1 expression in vivo in tumors.^433^

Recent approaches to deliver BH3-mimetics more safely and more selectively to the targeted tumor cells relied on alternative drug formulations or packaging. Nanoparticles can be used to encapsulate drugs, thus ensuring a more controlled release of the drug.^434^ Thereby, the coating of the nanoparticles may achieve a more tumor-targeted drug delivery and thus increase their therapeutic efficacy. This may be particularly important for combination treatments, which are associated with increased toxicity and unwanted side effects. The first evidence that this approach may be feasible for BH3-mimetics originates from a study with nanoencapsulated ABT-737 in combination with camptothecin.^435^ More recently, preclinical studies have shown that the combined treatment with venetoclax and S63845 was more efficacious and less toxic if either drug was administered in nanoformulation.^436^ In a more targeted approach, hyaluronic acid-coated nanocrystals were used to deliver venetoclax to CD44-expressing breast cancer cells,^437^ highlighting that receptor-ligand interactions can be exploited to selectively deliver BH3-mimetic containing nanoparticles to the intended cell populations.

Due to its function in platelet survival,^277^ packaging into nanoparticles may be particularly important for BH3-mimetics targeting BCL-X_L_. To this end, the dual BCL2/BCL-X_L_ inhibitor AZD4320 has been further developed as a PEGylated poly-lysine dendrimer conjugate.^438^ With a size of only 7–15 nm, the resulting dendrimer conjugate, AZD0466, is a small nanoparticle with good tumor penetration ability and a favorable therapeutic index.^439^ A first-in-human clinical trial indicated good tolerability of AZD0466 in patients with advanced solid tumors (NCT04214093). Further clinical development of AZD0466 was investigated in the phase I/II NIMBLE trial for AML and ALL patients (NCT04865419) as well as NHL patients (NCT05205161). However, both studies were terminated in 2023 due to an overall lack of significant clinical benefit.^440^

A different approach to delivering BH3-mimetics to the intended tumor cells may lie in the development of antibody-drug conjugates. To this end an antibody against B7H3 (mirzotamab) has been linked with the BCL-X_L_ inhibitor clezutoclax resulting in ABBV-155. ABBV-155 has been in clinical trial (NCT03595059) since 2018 in patients with advanced lung cancer. Preliminary results presented in 2022 and 2023 indicate that thrombocytopenia due BCL-X_L_ inhibition in platelets was not observed upon treatment with ABBV-155, suggesting that an antibody-targeted approach may indeed circumvent unwanted side effects of BCL-X_L_ inhibition.^441,442^ However, in this heavily pre-treated patient cohort, ABBV-155 also did not show anti-tumor activity, and this molecule has now been discontinued by Abbvie. More promising results were obtained by ABBV-637, an EGFR-targeted antibody coupled with a BCL-X_L_ inhibitor, which displayed some activity in a phase I study in R/R lung cancer patients (NCT04721015).^443^

### Strategies targeting the pro-apoptotic BCL2 proteins to prevent apoptosis

Not only too little but also too much apoptosis can contribute to disease. In this regard, strategies that prevent cell death by interfering with the BCL2 family may also hold therapeutic promise. Hence, small molecules inhibiting pro-apoptotic BAX activity such as MSN-50 and MSN-125 have emerged.^444^ Moreover, these compounds have already been reported to exert neuroprotective actions against glutamate-induced toxicity,^444^ a process driven by pro-apoptotic BAX.^445^ However, MSN-50 and MSN-125 also inhibit BAK. More recently, BAI1 has been designed as a novel, selective BAX-inhibiting molecule.^446^ Hence, such BAX-selective inhibitors could be attractive compounds to preserve neuronal survival.

## Targeting BCL2 proteins beyond cancer

### Neurodegenerative diseases

#### BCL2 proteins in Alzheimer’s disease

The BCL2 family of proteins plays a crucial role in neuronal survival and apoptosis regulation within the central nervous system (CNS) and hence it is not surprising that BCL2 proteins are implicated in numerous neurological diseases including Alzheimer’s disease (AD).^447,448^ AD is characterized by the accumulation of amyloid plaques consisting of amyloid β (Aβ). BCL2 proteins are downregulated in AD, whereby Aβ plaques can evoke an imbalance in the protein levels of BCL2 family members.^449,450^ In vivo exposure of brains or ex vivo treatment of hippocampal slides with Aβ oligomers results in a decrease in BCL2 proteins and an increase in BIM, thereby triggering the activation of BAX and neuronal death. BAX inhibition either using peptide-based approaches or gene therapy could prevent the neuronal cell death induced by oligomeric Aβ.^450^ Thus, in the context of AD, selective BAX inhibitors could be promising to sustain the survival of neurons and thus potentially delay cognitive decline.^451^

Beyond the inhibition of BAX, an upregulation of BCL2 protein levels in neurons has been explored as a neuroprotective strategy. Overexpressing of anti-apoptotic BCL2 in the postmitotic neurons of an AD mouse models reduced the activation of caspase 9 and caspase 3, thereby limiting the caspase-mediated cleavage of tau and the formation of neurofibrillary tangles.^452^ Furthermore, the neurons of these mice showed increased intracellular accumulation of amyloid precursor protein, thus restricting the formation of Aβ plaques. Importantly, AD mice overexpressing BCL2 displayed improved memory retention, thereby delaying the decline in cognitive function. This neuroprotective function of BCL2 upregulation preceded neuronal cell death, suggesting additional mechanisms are in play beyond BCL2’s canonical anti-apoptotic function (see below). Moreover, a correlation between increased expression of BCL2 in brain regions rich in Aβ plaques and neuroprotection was observed in an amyloid precursor protein transgenic mouse model for AD.^453^ This study further supported the neuroprotective functions of BCL2, which reduced the caspase 3-mediated cleavage of amyloid precursor protein and subsequently prevented the formation of extracellular Aβ plaques.^454^

However, the neuroprotective properties of BCL2 in AD are not limited to BCL2s canonical function in apoptosis, but also extend to the non-canonical role in regulating Ca^2+^ homeostasis. Over the years, it has become clear that deranged neuronal Ca^2+^ signaling is a very early hallmark of AD pathology, thereby driving pathogenesis.^455,456^ Hence, strategies to normalize or preserve Ca^2+^ signaling hold therapeutic promise to delay the deleterious impacts of AD progression.^457–459^ At the mechanistic level, both excessive Ca^2+^ release through IP_3_Rs^460–462^ and ryanodine receptors,^463–468^ potentially related in part to BCL2 downregulation, have been implicated in AD. Genetic or pharmacological inhibition of these channels could provide neuroprotection. Hence, BCL2, which inhibits both IP_3_Rs and ryanodine receptors, is an attractive target to suppress deranged Ca^2+^ signaling in AD.^72,469^ Moreover, endogenous ryanodine receptors/BCL2 complexes have been identified in neuronal samples,^72^ though whether those are dysregulated in AD awaits further validation. In any case, strategies that elevate BCL2 protein levels may provide neuroprotective effects through the targeting of ryanodine receptors by BCL2.^470^ Adeno-associated viral vectors expressing BCL2 proteins stereotactically injected in the brain of AD mice exerted synaptoprotective and amyloid-protective effects. Furthermore, overexpression of BCL2^K17D^, a BCL2 mutant that fails to inhibit IP_3_Rs but still inhibits ryanodine receptors, retained neuroprotective properties. These results suggest that inhibition of deranged ryanodine receptor channels in AD likely contributes to the neuroprotective role of BCL2 thereby inhibiting excessive Ca^2+^ responses in AD.^470^ However, since the molecular determinants in BCL2 responsible for ryanodine receptor binding remain elusive, BCL2 mutants that are defective in ryanodine receptor binding are not yet available to firmly establish ryanodine receptors as the neuroprotective targets of BCL2.

Finally, as an alternative to direct overexpression models of BCL2, several therapeutic drugs can increase BCL2 protein levels and can thus be implemented to improve AD outcomes. in this regard, drugs currently used for the treatment of asthma, such as ibudilast, montelukast, and pranlukast, can prevent BCL2 downregulation and improve Aβ-induced memory impairment.^471–473^

#### BCL2 proteins in Parkinson’s disease

Increasing evidence supports a role of dysregulated lysosomal function as an early feature in Parkinson disease (PD), and more specifically impaired lysosomal membrane permeabilization (LMP).^474^ The role of BAX/BAK in LMP and autophagic cell death,^475,476^ and its functional link to PD-linked lysosomal dysregulation has become clearer in recent years. Interestingly, BAX inhibition is able to prevent LMP, restore lysosomal levels and prevent accumulation of undegraded autophagosomes both in vitro and in vivo.^477^ Besides lysosomal defects, PD is characterized by degeneration of dopaminergic neurons in substantia nigra pars compacta (SNpc) and the loss of nerve fibers in the striatum. In experimental settings, neurotoxin 1-methyl-4-phenyl-1,2,3,6-tetrahydropyridine (MPTP) is used to replicate a PD-like syndrome by damaging SNpc dopaminergic neurons, mimicking the loss of these neurons.^478^ BAX is highly expressed in the SNpc and involved in degeneration of SNpc dopaminergic neurons in PD. Additionally, mice lacking BAX become resistant to MPTP-induced neurodegeneration.^479^ While MPTP administration increased BAX levels, a reduction in BCL2 protein levels was observed, thereby causing excessive BAX to exist free from neutralization by BCL2.^479^ Consistently, overexpression of BCL2 can also protect against MPTP-induced neurodegeneration.^480,481^

#### BCL2 in amyotrophic lateral sclerosis

The protein levels of different BCL2 proteins are altered in motor neuron disease-amyotrophic lateral sclerosis (ALS), which is characterized by excessive ER stress and disturbed protein homeostasis. Similarly to PD, BCL2 protein levels are reduced in ALS while BAK levels are increased.^482^ Familial forms of ALS are caused by mutations in superoxide dismutase-1 (SOD1). In SOD1G93A mice, neurodegeneration was prevented by overexpression of BCL2 and by delaying caspase activation, highlighting the importance of apoptosis in ALS.^483^ On the other hand, deletion of BAX improved the lifespan of SOD1G93A mice.^484^ Also, BIM targeting has been proposed as a potential strategy for ALS as upregulation of BIM in an ALS mouse model has been observed. By removal of BIM, the lifespan of the ALS mice was increased,^485^ highlighting the potential of targeting apoptosis as a potential therapeutic strategy for the treatment of ALS. However, so far this approach has only been tested in preclinical models, and no clinical trials are currently exploring whether this strategy may be translated to human disease.

### Autoimmune disorders

Given their central role at regulating immune cell survival, BCL2 proteins may also be implicated in autoimmune diseases characterized by insufficient apoptosis of autoreactive immune cells. Initial studies in mice indicated that loss of BIM or overexpression of BCL2 leads to the production of antinuclear autoantibodies, resulting in autoimmune disease resembling Systemic Lupus Erythematosus (SLE).^156,486^ In humans, SLE is associated with high levels of T cell-dependent antinuclear autoantibodies and systemic inflammation. Evidence for the role of BCL2 in SLE was provided by a study showing high BCL2 expression in circulating lymphocytes in SLE patients.^487–489^ In addition, BCL2 polymorphisms were found to be associated with the pathogenesis of SLE.^490^ In B cells derived from SLE, reduced induction of BCL-X_L_ and MCL1 levels were observed upon in vitro stimulation as compared to healthy control cells,^491^ which may indicate a higher functional importance of BCL2 also in B cells in SLE patients. These data suggest that targeting of BCL2 may represent a promising therapeutic strategy in SLE, and animal models of SLE treated with ABT-737 showed encouraging results.^492^ Based on these pre-clinical data, venetoclax has recently been tested in a phase I study in 73 women with SLE (NCT01686555). In these patients, venetoclax was well-tolerated and induced a concentration-dependent reduction in lymphocyte counts, especially in disease-relevant B cell subsets, highlighting the potential of venetoclax for the treatment of autoimmune diseases.^493–495^

Apart from SLE, a potential application of BH3-mimetics may be in the gastrointestinal autoimmune Crohn’s disease. Here, chronic inflammation is driven by hyperproliferating T cells with elevated BCL2 expression.^496,497^ BCL2 may also play a role in the associated intestinal fibrosis driven by activation of fibroblasts, which is linked to BCL2 and can be prevented by treatment with ABT-737 or ABT-263.^498,499^

More controversial is the role of BCL2 proteins in other autoimmune diseases including Type 1 diabetes mellitus (T1D).^500^ T1D is generally characterized by immune-mediated apoptosis of the β cells of the pancreas, resulting in insulin deficiency and chronic hyperglycemia. A recent study has indicated that in T1D, a subset of β cells acquires a senescence-associated secretory phenotype (SASP) associated with increased BCL2 expression.^501^ Treatment with senolytic BH3-mimetics (see below) may eliminate these senescent cells and prevent immune-mediated β cell destruction, indicating a new treatment strategy for T1D. In summary, although several studies indicate a potential for BH3-mimetics like venetoclax in different autoimmune diseases, there is currently no clinical development in this area.

### Ageing and senescence

The BCL2 proteins have been shown to play a major role in the survival of senescent cells.^502^ Cellular senescence was discovered after observing that human fetal cells only engaged in 40-60 replicative cycles and then ceased to divide any further.^503^ This number of divisions marks the replicative capacity of a cell and was eventually known as Hayflick’s limit, after which replicative senescence is induced. The mechanism underlying this phenomenon was later discovered to be the progressive shortening of telomeres with each mitotic division, which happens in all cells except those that express the telomerase enzyme, capable of telomere extension.^504^ In order to continue to grow beyond their natural numbers of division, cancer cells must adapt strategies to overcome and evade cellular senescence.^505,506^ The accumulation of senescent cells has also been found to play an important role in ageing and age-related diseases.^507^ Thus, preventing the accumulation of senescent cells has become a strategy with a strong potential to foster healthy ageing and ameliorate the prognosis of diseases such as diabetes, AD or cancer itself.^508^

This is the goal of a new class of therapeutics called senolytics that target senescent cells by selectively killing them.^508,509^ Early senolytics were discovered utilizing a combined hypothesis-driven and bioinformatic approaches, focusing on natural compounds or repurposing drugs already in use for other applications.^508^ Dasatinib, a pan-tyrosine kinase inhibitor approved for the treatment of myeloid leukemia, and quercetin, a naturally occurring flavonoid that may directly or indirectly inhibit BCL-X_L_, were two of the fist senolytics to be discovered, and are often applied together.^508^ This combination has not only been shown to improve physical function and increase the lifespan in aged mice,^510^ but also to reduce the presence of senescent cells in a phase I clinical trial in patients with diabetic kidney disease.^511^

These and other observations led to the study of BH3-mimetics as potential senolytics. Similar to cancer cells, senescent cells are primed for death but protected from apoptosis by the overexpression of BCL2 proteins.^512^ Indeed, senescent cells share many features with cells undergoing apoptosis, such as expression of p53, elevation of ROS or changes in mitochondrial physiology, along with consistent overexpression of BCL-w, BCL-X_L_, and BCL2.^513^ In addition, it has recently been discovered that minority MOMP (meaning MOMP occurring in a subset of mitochondria) is associated with cellular senescence.^514^ Although the factors that determine whether a cell will undergo apoptosis or senescence after damage are not fully understood, the BCL2 family of proteins seems to play an important role in these cell fate decisions, with expression of pro-survival BCL2 proteins favoring the induction of senescence over cell death.

ABT-737 was one of the first BH3-mimetics reported to exhibit selective pro-apoptotic activity in different senescent cell types.^513^ As a result of this, navitoclax is now widely used as a senolytic compound in pre-clinical research.^515^ A1331852 has also been found to have senolytic properties, which suggests that BCL-X_L_ may be the key protective protein in certain senescent cells.^358^ It has been shown to reduce senescence in cholangiocytes dependent on BCL-X_L_ and activated stromal fibroblasts, reducing liver fibrosis in vivo in MRD2^-/-^ mice.^516^ However, the senolytic activity of A1331852 and venetoclax is not as potent as that of navitoclax, suggesting that co-inhibition of BCL2, BCL-w and BCL-X_L_ may be more effective than BCL2 inhibition alone. Although BH3-mimetics seem to be some of the strongest senolytics known, with many potential clinical applications,^517^ the mixed response of different senescent cells to them will likely prevent their widespread use. This is probably due to the large heterogeneity of the senescent phenotype, which is still poorly understood and may include dependence on other pro-survival signals. Also, their off-target effects and toxicity may also limit their benefits as senotherapies.^515^ Nevertheless, BH3-mimetics are currently being used as part of an effort to design more targeted second-generation senolytics, which may change the way these drugs are delivered to their targets and improve their effects.^518,519^

## Future perspectives

### BCL2 inhibitors: can venetoclax be bettered?

It is said that “imitation is the sincerest form of flattery”, which means that if someone is copying your work, you must be doing something right! The combination of the transformational clinical activity in some hematologic malignancies and the concurrent absence of major toxicities of venetoclax has spawned an increasing number of “imitators” (Table 1 and Fig. 5e).

Venetoclax not only clinically validated the choice of BCL2 family proteins as therapeutic targets for cancer but also demonstrated the potential efficacy of specifically inhibiting protein-protein interactions. Moreover, the careful modeling of venetoclax to the BH3 binding groove of BCL2 made it appear unlikely that BCL2 inhibitors of improved binding and perhaps efficacy could be obtained. However, the development of sonrotoclax and lisaftoclax in particular, indicated that this may be the case, reflecting structural flexibility within the BCL2 BH3 binding groove. How far such improvements of binding of BCL2 inhibitors to BCL2 can be driven with the aid of modern computational modeling (Alphafold3 etc) remains to be determined.^520^

Improved binding of BCL2 inhibitors to BCL2 may not be without problems. Like all BCL2 family proteins, BCL2 is broadly expressed in normal tissues, and the lack of severe on-target toxicities with venetoclax reflects the inherent resistance of most normal tissues to BCL2 inhibition. Whether improved BCL2 binding will alter the toxicity profiles remains to be determined, although initial clinical data appear promising. One unanticipated consequence of BCL2 inhibition in some patients with CLL treated with either venetoclax or sonrotoclax is the acquisition of biallelic *BAX* mutations in normal myeloid cells.^323,521^ Their possible clinical significance remains unknown and will require careful monitoring. Biologically, they are fascinating since they would be anticipated to result in a profound apoptotic block and yet have not been detected in lymphoid malignant cells. In contrast, 17% of patients with AML treated with venetoclax exhibited various *BAX* mutations, pointing to a special role of BAX in myeloid cells.

With the emergence of acquired resistance in CLL or AML patients treated with venetoclax, one potential advantage of other BCL2 inhibitors may lie in their slightly different binding affinities to the individual pockets within the hydrophobic groove. However, resistance to venetoclax is not only mediated by point mutations within the hydrophobic groove, but also by the induction of other anti-apoptotic BCL2 proteins like MCL1 or BCL2A1.^276,522,523^ In contrast to MCL1, there is still no high-affinity inhibitor for BCL2A1.^77,524^ Overall, the hydrophobic groove of BCL2A1 is very similar to that of MCL1, but displays a unique Cys55 residue that may enable covalent binding of thiol-containing inhibitors.^525–528^ Further development of small molecule inhibitors of BCL2A1 may give rise to compounds able to counteract venetoclax resistance in selected patients. Given that BCL2A1 knockout mice are viable with only a minor hematological phenotype,^149^ inhibition of BCL2A1 may be associated with a favorable toxicity profile compared to MCL1 inhibitors.

### Novel applications of venetoclax

Since BH3-mimetics are designed to act downstream or independently of p53, a puzzling clinical observation was that despite initial responses, CLL or AML patients with mutated p53 are more likely to relapse early upon venetoclax treatment, indicating that functional p53 is required for durable responses to venetoclax.^529–531^ A molecular explanation for this observation was provided by the finding that BH3-mimetic induced MOMP activates p53 in a feedforward loop involving the release of mitochondrial DNA followed by cGAS/STING activation.^532^ Identification of this additional pathway also opens up the exciting possibility to intensify the responses to venetoclax in p53 mutated malignancies using STING agonists, and clinical trials investigating this combination are eagerly awaited.

### Development of novel formulations and delivery methods

While a multitude of combination treatments are currently being investigated mainly in preclinical studies, it remains to be seen whether these can increase tumor cell killing without simultaneously increasing on-target toxicities. In regards to tumor cell-selective delivery methods, both the development of nanoformulations and antibody-drug conjugates holds the potential to selectively deliver BH3-mimetics to malignant cells. To further increase tumor selectivity, these approaches could be combined with the delivery of a PROTAC molecule instead of a BH3-mimetic, thus incorporating another selection criterion. These approaches are still in their infancy, but with the rapid advances in these fields and the high importance of MCL1 and BCL-X_L_ as therapeutic targets, it is likely that progress will be rapid.

### Tailoring BCL-X_L_ or MCL1 inhibitors for selective tumor cell killing

A plethora of preclinical studies^533^ has established that for many tumor cells, the combined inhibition of BCL-X_L_ and MCL1 may be highly synergistic and efficiently kill tumor cells, thus paving the way for a potential chemotherapy-free treatment of many cancer types. Based on their significance in solid tumors, selective inhibition of MCL1 or BCL-X_L_ in cancer cells represents the “Holy Grail” in the field of BH3-mimetics. However, with the toxicities reported particularly for selective MCL1 inhibitors and the subsequent termination of multiple candidates, highly potent BH3-mimetics may not be the right way forward. Potential solutions could be provided either by combination treatments allowing for a subtoxic dosage regiments or by tumor cell-selective delivery of BH3-mimetics. An alternative approach for MCL1 is to target indirectly, either by selective protein degradation or suppression of mRNA synthesis. For example, the short half-lives of MCL1 mRNA and protein may permit selective suppression by CDK9 inhibition.^534,535^ MCL1 protein may also be selectively targeted for proteasomal degradation by the CD79B antibody-drug conjugate, polatuzamab vedotin.^308^

### Therapeutic application beyond cancer

Mechanistic understanding of the BCL2 family and their role in regulating cellular survival also highlight potential applications beyond cancer. Currently developed treatment options may directly be applicable to some autoimmune diseases like SLE, where preclinical experiments and early clinical studies indicate some activity of BH3-mimetics. However, how BH3-mimetics may compare to other immune-dampening drugs currently applied for autoimmune diseases needs to be carefully assessed and toxicities need to be monitored. Besides autoimmune diseases, BH3-mimetics may serve as senolytic drugs, and thus improve some age-related diseases. Although studies on senolytics are still in their infancy, these may be of huge impact in an aging society. For the treatment of neurodegenerative diseases on the other hand, a potential therapeutic approach would be to modulate the BCL2 family in such a way that neurons are protected from pathological cell death. The advancements in technologies particularly for gene transfers may open up new possibilities to maintain neuronal survival and mitigate cell loss associated with neurodegenerative diseases like AD.

## Conclusions

BCL2 represents a therapeutic target for some hematological malignancies, but not for most solid tumors. Venetoclax is highly successful in all CLL and some AML patients, but for other hematological malignancies most notably DLBCL, clinically actionable biomarkers are urgently required to define patient populations most likely to benefit from BCL2 inhibition. In their absence, the empirical addition of BCL2 inhibitors to therapeutic regimens may add to physical and financial toxicities for no additional clinical benefit.^536^ As we have argued previously, some form of functional assessment is necessary to match precision medicines to specific malignancies.^537^ In B cell malignancies, the field is moving rapidly towards “chemotherapy-free” combinations of precision medicines, as illustrated by the recently-reported ViPOR study, which utilized obinutuzumab, ibrutinib, venetoclax, lenalidomide, and prednisone in patients with R/R LBCL in a small single-center phase II study.^536^ This regimen was for the most part well tolerated and produced a complete remission rate of 38% in this difficult-to-treat group of patients. However, empirically, one can already consider potential improvements such as the addition of polatuzumab vedotin and replacing obinutuzumab with CD20xCD3 bispecific antibodies.^538^ It should be noted that none of these precision medicines are tumor-specific and in combination, may result in more severe long-term immunosuppression than regular chemotherapy, which, especially during viral pandemics, can have fatal consequences.

In solid tumors, both MCL1 and BCL-X_L_ represent highly promising therapeutic targets, but current approaches to inhibit these proteins are limited by on-target toxicities and the clinical development of several highly promising compounds has been terminated. Novel approaches to deliver BH3-mimetics or similar compounds safely to the targeted cell types are currently being explored, and with the technological advances in nanotechnology and protein engineering, it appears to be just a matter of time before successful strategies will be translated into clinical applications. Therefore, targeting of the BCL2 protein family is entering an exciting new era, and multiple new applications for modulating the activity of BCL2 proteins selectively in diseased tissues are foreseeable even beyond the treatment of cancer.
